# Exosomal lncRNA XR_001793654.1 in human cardiac explant-derived alleviates atrial fibrillation via abolishing the miR-107-3p-mediated KLF13 inhibition

**DOI:** 10.3389/fcell.2025.1694467

**Published:** 2025-11-07

**Authors:** Yanxiao Liang, Dongyu Li, Huishan Wang, Yuan Tian

**Affiliations:** 1 Department of Cardiac Surgery, Shengjing Hospital of China Medical University, Shengyang, China; 2 Department of Cardiovascular Surgery, General Hospital of Northern Theater Command, Shengyang, China; 3 Department of Laboratory Medicine, Shengjing Hospital of China Medical University, Shengyang, China

**Keywords:** atrial fibrillation, extracellular vesicles, lncRNA XR_001793654.1, miR-107-3p, KLF13

## Abstract

**Background:**

Atrial fibrillation (AF) is a type of arrhythmia that occurs in the upper part of the heart. Extracellular vesicles (EVs) released by human cardiac explant-derived cells (CDCs) contain bioactive cargos that may function as diagnostic indicators or therapeutic candidates for AF. The lncRNA XR_001793654.1 has been identified as a putative modulator in AF pathogenesis.

**Methods:**

Human CDCs were derived from left atrial appendages collected during cardiac surgery. Then, EVs were isolated from cultured CDCs using ultracentrifugation. XR_001793654.1 expression was quantified in EVs. The relationship between XR_001793654.1, miR-107-3p, and KLF13 was identified.

**Results:**

XR_001793654.1 was upregulated in EVs from human CDCs. Systemic delivery of these EVs *in vivo* diminished atrial fibrosis and hypertrophy, with concurrent suppression of inflammatory cell accumulation and pro-inflammatory cytokine release. Mechanistically, EV-associated XR_001793654.1 served as ceRNA, sequestering miR-107-3p and thereby alleviating its inhibitory regulation of KLF13 expression. Elevation of KLF13 was essential for the observed cardioprotective outcomes.

**Conclusion:**

In conclusion, human CDC-derived EV containing XR_001793654.1 alleviates atrial fibrosis and AF through neutralizing miR-107-3p-mediated downregulation of KLF13. These findings offer new perspectives on AF molecular mechanisms and emphasize XR_001793654.1 as a promising intervention target.

## Highlights

- XR_001793654.1 is the most highly expressed transcripts within human CDC-derived EVs.
- Intramyocardial injection of human CDC-derived EVs attenuates AF.
- XR_001793654.1 relieves AF via miR-107-3p/KFL13 axis.

## Introduction

Atrial fibrillation (AF), a prominent cardiac dysrhythmia, arises from a widespread and disorganized pattern of electrical impulses within the atria, which suppresses the normal sinus mechanism (Vinciguerra et al., 2024; Linz et al., 2024). This condition is prone to result in significant complications regarding morbidity and mortality, particularly when hemorrhagic or thromboembolic complications occur, imposing a high global burden of disease (Joseph et al., 2021). Existing approaches to address AF primarily concentrate on catheter ablation, anticoagulant therapy, antiarrhythmic medications, and operative procedures. Nevertheless, the prevalence and burden of AF continue to rise. Identifying molecular and cellular regulators that influence AF onset and progression is essential for advancing both preventive and therapeutic strategies.

Extracellular vehicles (EVs), which include exosomes and microvesicles, etc., engage in interactions with target cells and transfer their active content, such as RNAs, proteins, and lipids. The identification of EVs offers new insights into the intricate mechanisms underlying AF and suggests possible biomarkers for evaluating the disease severity or predicting prognosis (Shaihov-Teper et al., 2021; Parent et al., 2023; Parent et al., 2024). EVs may be purified from conditioned media of cardiac explant-derived cells (CDCs) for immediate utilization as an acellular therapeutic modality (Andriolo et al., 2018). CDCs are CD45^−^ CD105^+^ cells expanded directly from atrial tissue biopsies for clinical (Parent et al., 2023; Milano et al., 2020; Barile et al., 2018; Mount et al., 2019). In the recovery model after myocardial infarction, EVs can be injected into the myocardium once. During the procedure of open-chest surgery, this approach not only can alleviate the inflammatory response but also enables local fibroblasts to resist the stimulatory factors for fibrosis (Parent et al., 2024). Within our current investigation, we assessed the capacity of the therapeutic potential of human atrial explant-derived CDC-EVs in attenuating AF within a rabbit model.

Long noncoding RNAs (lncRNAs) exert regulatory effects on the onset and progression of numerous diseases (Nemeth et al., 2024; Ferrer and Dimitrova, 2024). An expanding body of research in the field of cardiovascular biology has indicated that distinctly expressed lncRNAs exert a pivotal function in the pathogenesis of numerous diseases, such as heart failure, cardiac hypertrophy and AF (Yang et al., 2021; Lin et al., 2021; Le and Nhu, 2023). It is recognized that the pulmonary vein-left atrial junction and the left atrium are significant sites of origin for electrophysiologic abnormalities in AF (Wijesurendra and Casadei, 2019). Electrical triggering remains a key initiating factor for patients who have not yet reached the stage of permanent AF. This implies that interventions at the site of origin of the electrophysiologic abnormalities can markedly lower the incidence of postoperative complications of AF and, consequently, enhance the electrical and structural remodeling of the condition (Nattel et al., 2020). Therefore, additional investigation into the expression and regulatory mechanisms of cardiovascular disease-related lncRNAs will aid in uncovering novel treatment methods for AF.

LncRNAs can regulate the expression of microRNAs (miRNAs) by adsorbing them and binding to their specific sequences, similar to the adsorption function of a “sponge.” Therefore, crucial regulatory effects are exerted by LncRNA in many diseases (Nemeth et al., 2024; Ferrer and Dimitrova, 2024). More and more evidence has manifested that differentially expressed lncRNAs play an indispensable role in many cardiovascular diseases such as heart failure, heart hypertrophy and AF (Le and Nhu, 2023). Here, we identified that XR_001793654.1 may function as a biomarker to indicate the development of AF due to its participation in fibrosis signaling pathways and oxidant stress. Through the use of the miRWalk database, it was predicted that XR_001793654.1 has a binding site with miR-107-3p. The interaction sites of miR-107-3p were also suggested in the 3′UTR of KLF13. KLF13, a cardiac transcription factor, is involved in cardiac development (Li et al., 2020; Huang et al., 2007). Therefore, it was hypothesized whether human CDC-derived EVs might transport XR_001793654.1, which could play a noteworthy role in AF development through modulating the miR-107-3p/KLF13 axis.

## Methods

### Rapid atrial pacing (RAP) rabbit model

This research has been approved by the Animal Experiment Ethics Committee of China Medical University. We strictly followed all the guidelines stipulated in the “Regulations on the Administration of Laboratory Animals”. Adult New Zealand white rabbits, weighing 2.8–3.4 kg and approximately 14 weeks of age, were sourced from Qingdao Kangda Biotechnology Co., Ltd., and housed in the Animal Experimental Center of the Northern Theater General Hospital, which is affiliated with China Medical University. The induction of the atrial fibrillation (AF) model in rabbits followed established protocols (Liu et al., 2016; Jiang et al., 2022). In this research, a surface electrocardiogram was conducted for each rabbit to ensure they were in sinus rhythm without any detectable cardiac arrhythmias prior to the intervention. The animals were sedated using lidocaine hydrochloride (dosed at 2–4 mg/kg). A cardiac pacemaker was implanted into a subcutaneous pouch on the rabbits’ dorsal side, with the pacemaker leads attached to the left atrium. Post-implantation of the cardiac pacemaker, penicillin was delivered intramuscularly for three consecutive days to avert infectious complications. For the AF group, a constant pacing frequency of 10 Hz (600 beats/min) was maintained for 7 days to induce the AF condition in the rabbit models. The sham group underwent identical procedures without pacing intervention. Following the surgical procedure, the animals were allowed a 1-week convalescence period. Detailed description of the experimental method about the atrial fibrillation induction model in the Supplementary Material (Supplementary Figures S1–S6; Supplementary Tables S1–S3).

### Cell culture and the isolation and identification of exosomes

Human CDCs derived from the left atrial appendages were procured with informed consent during cardiac surgeries, following a protocol sanctioned by the Ethics Committee of the Northern Theater General Hospital, which is affiliated with China Medical University. The CDCs were grown in a serum-free, xeno-free environment using NutriStem XF medium (Sartorius) in a stable 5% oxygen atmosphere (Mount et al., 2019). The conditioned media were produced by culturing cells for 48 h at a 1% oxygen level. The glucose content of the used culture medium increased, and 1% platelet lysate was added, which was called the Dulbecco-modified Ehrlich medium. EVs were harvested through a process of ultracentrifugation, which included spins at 10,000 g for 30 min and 100,000 g for 3 h. Subsequently, the purified EVs were visualized under a transmission electron microscope (TEM) and their characteristics were assessed through nanoparticle tracking analysis (NTA). THP-1 cells (TIB-202, ATCC) were cultivated in RPMI1640 medium enriched with 10% fetal bovine serum, followed by differentiation into macrophages using 100 nM phorbol 12-myristate 13-acetate (PMA) for 3 days.

### lncRNA adeno-associated virus infected atrial muscle in rabbit AF model

After the two electrode wires were sutured in the left atrium during the modeling process, the virus was randomly selected at 8 sites in the left atrium between the two stimulation electrodes, and 50 ul virus vector was injected diagonally into the muscle of the left atrium with a microsyringe. The virus titer was 2 × 10^12^ ug/mL (Tan et al., 2022).

To investigate the role of XR_001793654.1 in the rabbit AF model, the model rabbits were randomly divided into 2 groups: (1) the lncRNA-overexpressing adeno-associated virus group (AAV-XR_001793654.1, n = 6); (2) the lncRNA adeno-associated virus empty vector control group (AAV-NC, n = 6).

To explore the biological role of miR-107-3p in the pathogenesis of rabbit AF model, the model rabbits were randomly divided into 6 groups: (1) miR-107-3p overexpressing AAV group (miR-107 mimic, n = 3); (2) miR-107-3p overexpressing AAV empty vector control group (mimic NC, n = 3); (3) miR-107-3p inhibitor AAV group (miR-107-3p inhibitor, n = 3); (4) miR-107-3p inhibitor AAV empty vector control group (inhibitor NC, n = 3); (5) XR_001793645.1 overexpressed AAV + miR-107-3p overexpressing AAV group (AAV-XR_001793645.1+miR-107-3p mimic, n = 3); (6) XR_001793645.1 overexpressed AAV empty vector + miR-107-3p overexpressing AAV empty vector cotransmutation group (AAV-NC + mimic NC, n = 3).

### Dual-luciferase reporter assay

Wild-type (WT, wild-type) and mutant (MUT, mutant) gene sequences of XR_001793654.1 and KLF13 3′ UTR were chemically synthesized. Subsequently, the pmiR-RB-REPORT™ vector plasmid was subjected to restriction endonuclease digestion. The synthesized WT and MUT DNA fragments were then cloned into the pmiR-RB-REPORT™ vector, along with miR-107-3p mimics; this was performed to verify the binding interaction between XR_001793654.1, miR-107-3p, and KLF13 3′ UTR. The Dual-Luciferase Reporter Assay was performed to determine relative luciferase activity (Chen et al., 2021). The gene sequences are as follows:

XR_001793654.1-WT: TCCCTATGGAAGGGAAGTGCTGCU;

XR_001793654.1-MUT: TCCCTATGGAAGGGAAGCTTGATU;

KLF13-3′UTR-WT: GTCCTACAGGATGCTGCU;

KLF13-3′UTR-MUT: CTTTCCCTGGTAACGTAU.

### Western blot (WB)

The atrial tissue was ground and lysed using RIPA buffer. The total cellular proteins were separated and extracted. The total protein concentration was detected using the BCA method (BCA assay kit), and subsequent processing was carried out after meeting the requirements. First, the proteins to be detected were subjected to gel electrophoresis, separated by SDS-PAGE, and transferred onto a polyvinylidene fluoride (PVDF) membrane. Then, at room temperature, the membrane was blocked with 5% bovine serum albumin (BSA) for 1 h, mainly to prevent nonspecific protein binding sites. Next, the specific primary antibody was incubated with the membrane at 4 °C overnight, followed by incubation with horseradish peroxidase (HRP)-labeled goat anti-rabbit IgG or goat anti-mouse IgG at room temperature for 1.5 h. Subsequently, developing solutions were added to the membranes, and protein band intensities were analyzed using ImageLab software. Finally, the target bands were tested for their gray values, and compared with the gray value of the marker protein GAPDH. The obtained ratio was the relative protein expression level of the target gene.

### Statistical analysis

Quantitative data, including luciferase activity, protein expression levels, and other measurable parameters, were presented as mean ± standard deviation. Data analysis was conducted with SPSS v26.0 and GraphPad Prism v8.2 software. Statistical significance was determined using an unpaired T-test (for pairwise comparisons) or one-way analysis of variance (ANOVA) with Tukey’s multiple comparisons test or repeated measures ANOVA with Bonferroni *post hoc* test (for multi-group comparisons). P < 0.05 was considered statistically significant.

Detailed descriptions of the construction of lncRNA and miRNA adeno-associated virus (AAV), AAV *in vivo* infection, detection of cardiac electrophysiological indicators, and RNA sequencing (RNA-seq) are available in the Supplementary Material.

## Results

### Identification of human cardiac explant-derived cell-derived extracellular vesicles

**FIGURE 1:**
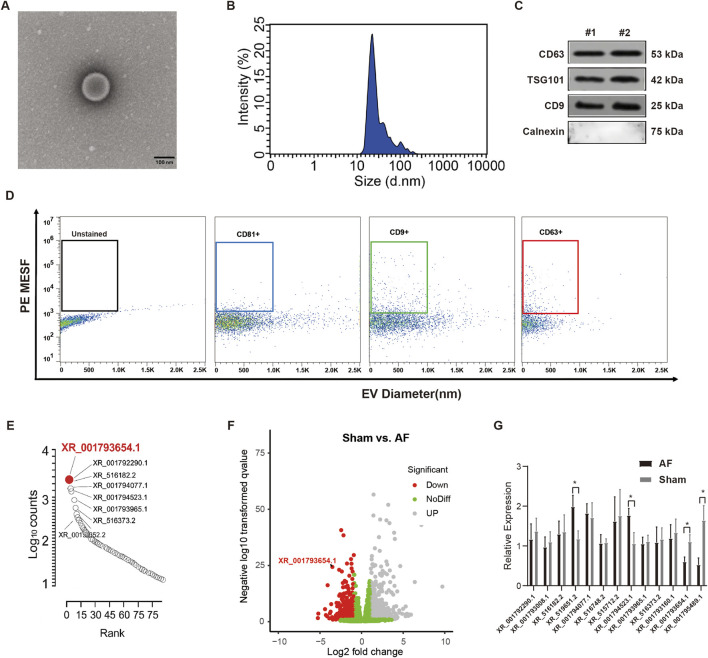
XR_001793654–1 exhibits high expression in the human CDC-derived EVs. **(A)** Transmission electron microscopy (TEM) for morphological analysis of EVs (scale bar = 100 nm). **(B)** Nanoparticle tracking analysis (NTA) for size distribution of EVs. **(C)** EV markers (CD63, CD9, TSG101) and the negative marker Calnexin were analyzed via Western blot. **(D)** Flow cytometric analysis of the size of vesicles and the expression of surface markers. **(E)** Quantification of lncRNA expression in human CDC-derived EVs. **(F)** Volcano plot of altered lncRNA expression in atrial fibrillation (AF) and sham rabbit models. **(G)** qRT-PCR analysis of atrial tissues from AF and sham rabbit models. *P < 0.05, #P < 0.01.

We first identified the isolated EVs from human CDCs. A TEM look into revealed that the human CDC-derived EVs exhibited a round or oval membranous morphology, consistent with the typical characteristics of EVs (Figure 1A). Their diameter ranged from 30 to 150 nm, as determined by nanoparticle tracking analysis (NTA) (Figure 1B). Western blot demonstrated that CD63, CD9, and TSG101 proteins were detected in the human CDC-derived EVs isolated from two patient samples, while Calnexin (an endoplasmic reticulum marker, indicating absence of cellular contamination) was absent (Figure 1C). Flow cytometry confirmed a significant presence of microparticles positive for typical EV markers CD9, CD63, and CD81 (Figure 1D). The EV cargo was rich in lncRNAs associated with AF, with XR_001793654.1 being the most abundantly transcribed (Figure 1E). Among the lncRNAs, XR_001793654.1 was the most highly expressed transcript. Furthermore, a total of 18,722 lncRNAs were detected via RNA-Seq, of which 55 transcripts were upregulated (Figure 1F). These lncRNAs were verified by qRT-PCR. The experimental results demonstrated that the expression of XR_001793654.1 was remarkably downregulated in the AF relative to the sham-operated group, indicating a potential association between XR_001793654.1 downregulation and AF pathogenesis (Figure 1G). The biological efficacy of EVs with lncRNA XR_001793654.1 has also been explored to demonstrate a dose-response relationship with increasing concentrations of EVs used for treatment (Supplementary Material).

### Intramyocardial injection of human CDC-derived EVs attenuates AF

**FIGURE 2:**
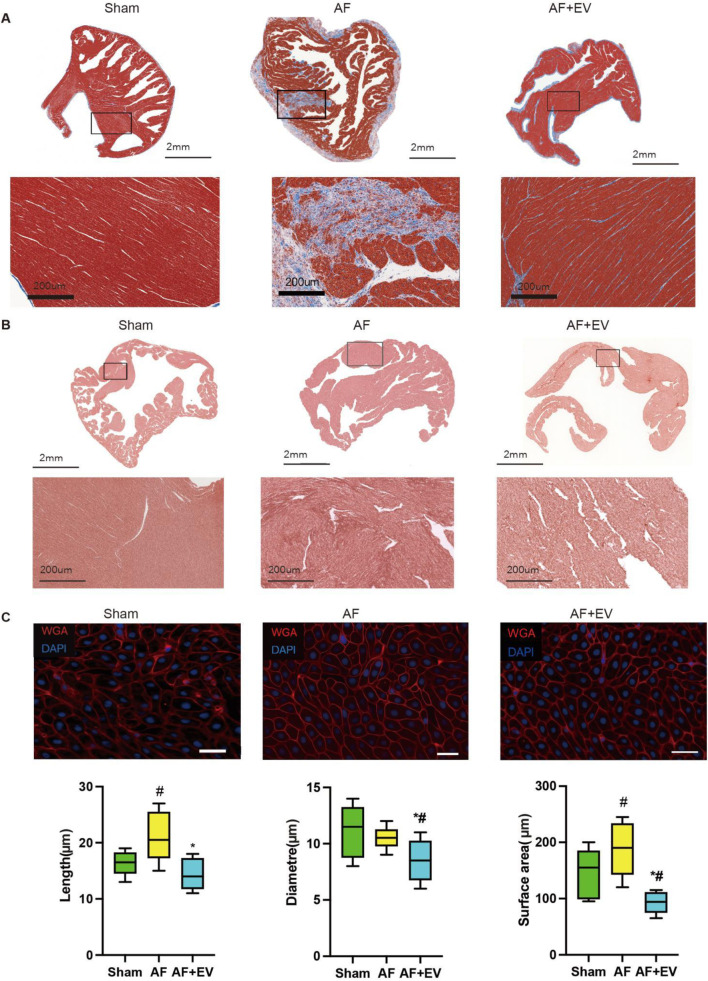
Intramyocardial injection of human CDC-derived EVs reduces the severity of atrial fibrillation (AF). **(A)** Masson’s Trichrome staining. Scale bar, 2 mm. **(B)** Picrosirius red staining. Scale bar, 2 mm. **(C)** Wheat germ agglutinin (WGA) and 4′,6-diamidino-2-phenylindole (DAPI) staining. Scale bar, 20 μm. *P < 0.05, #P < 0.01.

**FIGURE 3:**
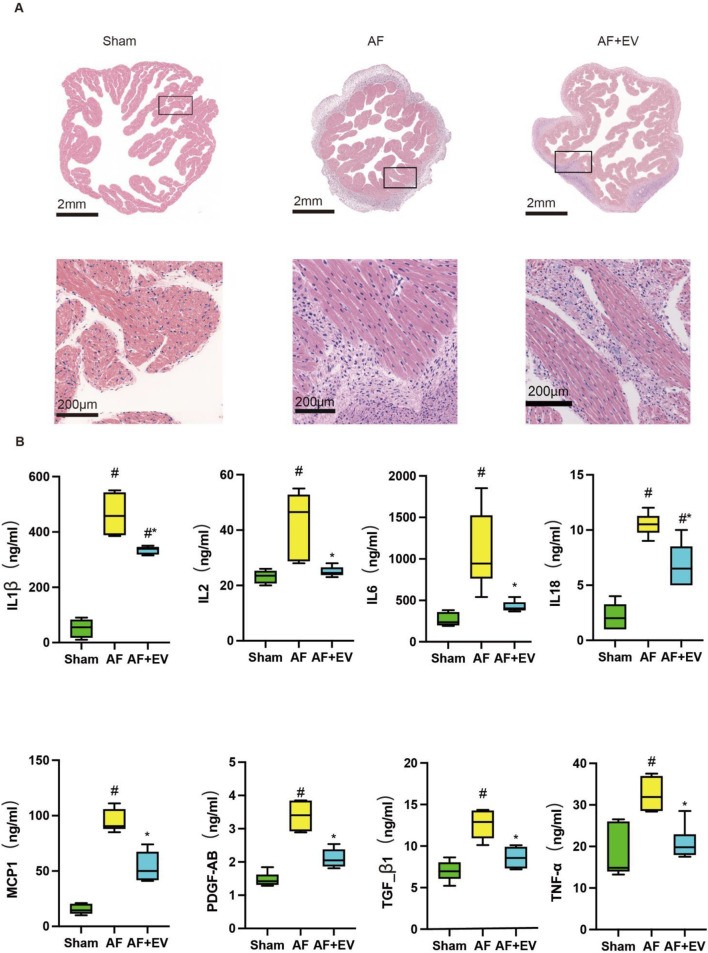
Intramyocardial injection of human CDC-derived EVs reduces inflammatory cell infiltration. **(A)** Hematoxylin and eosin (H&E) staining. Scale bar, 2 mm or 200 μm. **(B)** Effects of human CDC-derived EVs on the expression of inflammatory factors. *P < 0.05, #P < 0.01.

As shown in Figures 2A,B, Masson’s trichrome (for fibrosis detection) and picrosirius red staining (for collagen visualization) demonstrated that intramyocardial injection of human CDC-derived EVs attenuated atrial fibrosis in AF rabbits. Wheat germ agglutinin (WGA) of atrial tissue sections further confirmed that EVs reduced cardiomyocyte hypertrophy (Figure 2C). The effect of human CDC-derived EVs on inflammation was assessed through atrial histology, revealing a marked augmentation in inflammatory cell infiltration in AF rabbits, which was mitigated by EV treatment (Figure 3). This was evidenced by a decrease in proinflammatory cytokines (key mediators of atrial inflammation) in the atria of the rabbits in the treatment group. AF-induced increases of multiple proinflammatory cytokines, including IL-2, IL-1β, MCP-1, IL-6, IL-18, PDGF-AB, TGF-β1 and TNF-α. Intramyocardial injection of EVs blocked the increase of a large number of these cytokines (IL-2, IL-6, TNF-α, TGF-β1, and PDGF-AB) and reduced the elevation of others (MCP-1, IL-1β and IL-18).

**FIGURE 4:**
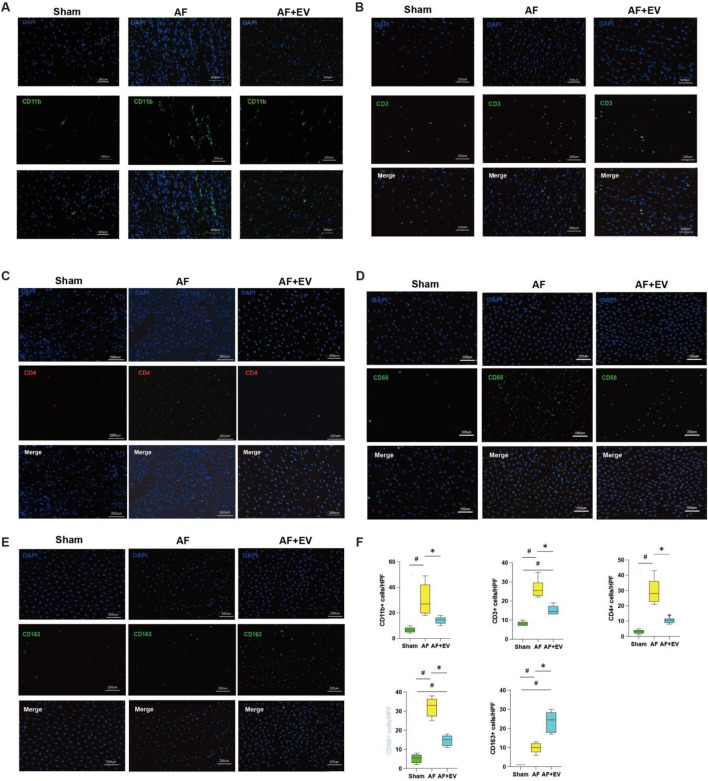
Intramyocardial injection of human CDC-derived EVs induces M2 macrophage polarization. Effects of human CDC-derived EVs on the number of neutrophils (CD11b+) **(A)**, cytotoxic T cells (CD3^+^) **(B)**, T helper cells (CD4^+^) **(C)**, pro-inflammatory M1 macrophages (CD68^+^) **(D)**, and anti-inflammatory M2 macrophages (CD163+) **(E)**. **(F)** Comparative statistical analysis between groups. *P < 0.05, #P < 0.01.

In order to clarify the underlying mechanism, the immune cell subsets within atrial tissues were evaluated 3 days post-surgery. AF elevated the number of neutrophils (CD11b^+^, a neutrophil marker), cytotoxic T cells (CD3^+^, a T cell marker), and T helper cells (CD4^+^, a helper T cell marker) in the atria. The intervention treatment with EVs remarkably reduced the recruitment level of these cells to baseline levels (Figure 4). AF also elevated the number of proinflammatory M1-type macrophages (CD68^+^) and anti-inflammatory M2-type macrophages (CD163^+^). EV treatment increased M2-type macrophages and decreased M1-type macrophages, suggesting a phenotypic switch towards an anti-inflammatory M2 phenotype, which may represent a key mechanism underlying the anti-inflammatory effects of EVs.

### XR_001793654.1 relieves AF via the miR-107-3p/KFL13 axis

**FIGURE 5:**
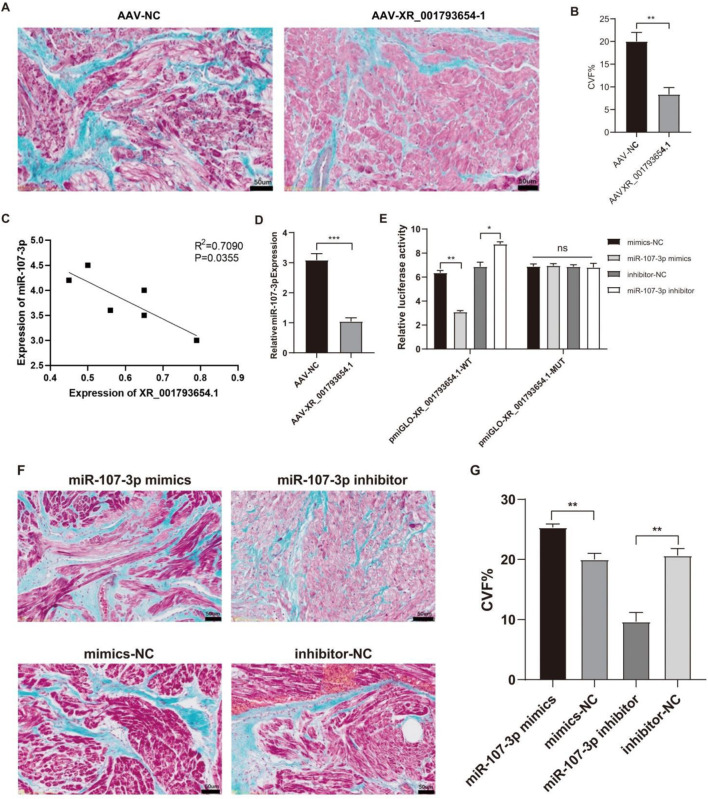
Overexpression of XR_001793654. 1 decreases left atrial fibrosis in a rabbit AF model. **(A)** Masson’s Trichrome staining. Scale bar, 2 mm. **(B)** Comparative statistical analysis of cardiac collagen volume fraction (CVF%). **(C)** Correlation between XR_001793654.1 and miR-107-3p. **(D)** Expression of miR-107-3p after AAV-XR_001793654.1 or AAV-NC transfection. **(E)** Dual-luciferase reporter assay. **(F)** Masson’s Trichrome staining. Scale bar, 2 mm. **(G)** Comparative statistical analysis of cardiac collagen volume fraction (CVF%). *P < 0.05, **P < 0.01.

As shown in Supplementary Table S2, compared with the AAV-NC group, LAD, LAVmax, LAVmin and other indexes in the AAV-XR_001793654.1 group after 4 weeks of stimulation were significantly decreased, while LAEF was significantly increased. Masson staining was performed on the left atrial samples of the AAV-XR_001793654.1 group and the AAV-NC group to observe the degree of left atrial muscle fibrosis (Figure 5A). Compared with the AAV-NC group, the degree of atrial fibrosis in the AAV-XR_001793654.1 group was significantly reduced (*P* < 0.01; Figure 5B). The above results indicate that the overexpression of XR_001793654.1 could effectively relieve the left atrial enlargement and left atrial dysfunction in the rabbit AF model.

Furthermore, the expression levels of XR_001793654.1 were inversely associated with those of miR-107 in the left atrial appendage tissues (Figure 5C). Following the forced expression of XR_001793654.1 within this model, qRT-PCR demonstrated a reduced expression of miR-107 in the AAV-XR_001793654.1-transfected group compared with the AAV-NC group (Figure 5D). A dual luciferase reporter gene assay verified the direct binding capacity of XR_001793654.1 to the miR-107 sequence, evidencing an interaction (Figure 5E). These results imply a negative correlation between XR_001793654.1 and miR-107-3p in the left atrial appendage tissue. Histological examination using Masson’s trichrome staining revealed that the fibrosis in the miR-107-3p inhibitor group was mitigated, whereas it was exacerbated in the miR-107-3p mimic group (Figures 5F,G). These results imply that the suppression of miR-107-3p can diminish left atrial fibrosis in the AF model of rabbit.

**FIGURE 6:**
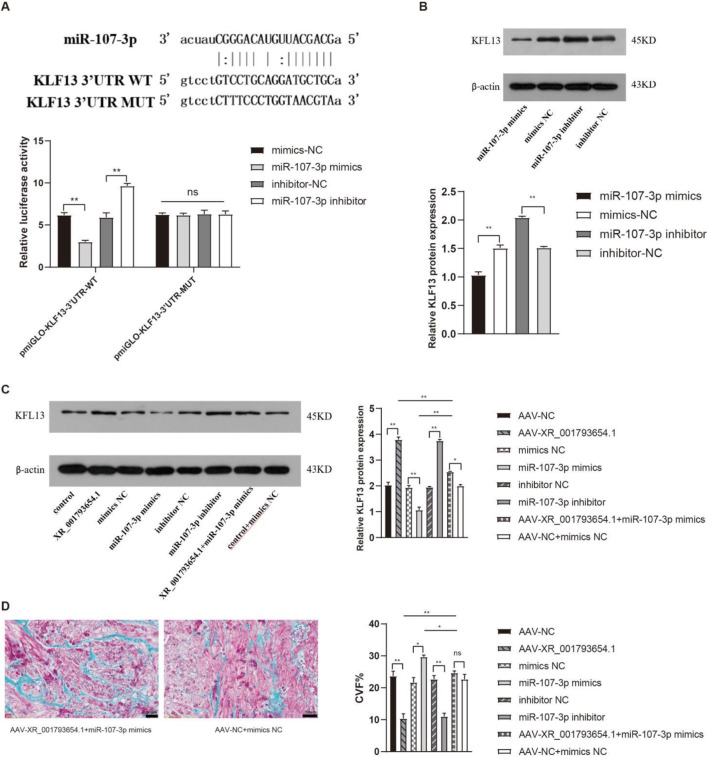
XR_001793654.1 modulates AF via the miR-107-3p/KLF13 pathway. **(A)** Dual-luciferase reporter assay confirming that the KLF13 3′UTR can bind to the miR-107-3p sequence. **(B)** Western blot analysis showing that KLF13 expression was downregulated in the miR-107-3p mimic group and upregulated in the miR-107-3p inhibitor group. **(C,D)** Western blot analysis and Masson staining of *in vivo* co-transfection experiments with XR_001793654.1-overexpressing AAV and miR-107-3p-overexpressing AAV in a rabbit AF model. *P < 0.05, **P < 0.01.

Bioinformatics analysis and sequence alignment revealed a putative binding site for miR-107 in the 3′UTR region of KLF13 (Figure 6A), hinting that KLF13 could serve as a downstream target of miR-107. Subsequent confirmation through a dual luciferase reporter gene assay established that the 3′UTR of KLF13 can bind to the miR-107 sequence. Western blot (WB) analysis indicated that the expression levels of KLF13 protein were reduced in the miR-107-3p mimic group and were elevated in the miR-107-3p inhibitor group (Figure 6B). The outcomes propose that miR-107-3p may modulate atrial fibrillation by regulating KLF13 expression.

Subsequent co-transfection experiments, introducing AAV overexpressing XR_001793654.1 and AAV overexpressing miR-107-3p into the rabbit atrial fibrillation model, were performed to elucidate their potential interaction. WB analysis showed that the overexpression of XR_001793654.1 resulted in an upregulation of KLF13 expression. Conversely, the co-transfection of miR-107-3p mimic abrogated the upregulatory effect of XR_001793654.1 overexpression on KLF13 (Figure 6C). Masson’s trichrome staining substantiated these findings, showing no statistically remarkable difference in fibrosis severity between the adeno-associated virus (AAV)-XR_001793645.1 + miR-107-3p mimic group and the AAV-NC + mimic NC group (Figure 6D). These results suggest that XR_001793654.1 modulates the expression of the target gene KLF13 by competitively binding to miR-107-3p, thereby influencing the pathogenesis of atrial fibrillation.

## Discussion

Previously, the cells isolated from cardiac tissue were capable of secreting and generating various substances, including miRNAs with anti-fibrotic and anti-inflammatory functions, as well as specific EV products of proteins (Parent et al., 2023; Parent et al., 2024). By directly injecting exosomes into the atrial wall during open-heart surgery, we observed a significant reduction in both the inflammation and fibrosis of the atrium. In this study, our results demonstrated that exosomes can effectively alleviate inflammation and fibrosis. Mechanistically, we noted alterations in the levels of inflammatory cytokines and tissue remodeling markers attributed to the cargo of lncRNA XR_001793654.1. To further validate the role of XR_001793654.1, we administered an *in vivo* injection of an AAV vector overexpressing XR_001793654.1 in a rabbit model of AF. The findings indicated that the overexpression of XR_001793654.1 improved left atrial function and significantly mitigated left atrial fibrosis in the rabbit AF model.

Considering that AF typically emerges 1–3 days after the surgery, therapeutic intervention must be implemented before the injury occurs. They should take effect during this crucial 1–3 days recovery period and avoid adversely affecting postoperative healing. Here, we investigated whether the injection of EVs into the atria could alleviate post-surgical inducible AF without performing surgery or using systemic medication. Atrial inflammation, which is critical in triggering AF, was reduced following intramyocardial injection of human CDC-derived EVs. This is consistent with previous studies showing the known anti-fibrotic effect of exosomes on ventricular remodeling after infarction (Parent et al., 2024; Hermann et al., 2022). The addition of exosomes reduced the sensitivity of atrial fibroblasts to inflammatory signals and alleviated the fibrotic process.

In this study, the hypothesis that intramyocardial injection of cardiac cell-derived EVs reduces postoperative AF incidence was tested. To verify this underlying mechanism, our results revealed that exosomes attenuate neutrophil infiltration, M1-type macrophage polarization, and proinflammatory cytokine production. These effects were validated via histological and cytokine array analyses. Mechanistically, exosomes are thought to prime atrial cells to restrict inflammatory signaling pathways. This mechanism aligns with findings from ventricular remodeling studies, which demonstrated that cardiac cell-derived exosomes alter the polarization state of resident and infiltrating macrophages (Fang et al., 2022). It also corroborates research emphasizing that inflammatory signals in atrial myocytes—key drivers of atrial structural remodeling and AF initiation—play a critical role in AF pathogenesis (Dobrev et al., 2023). This *in vivo* effect may be partially mediated by inhibiting proinflammatory cytokine release from resident cells.

Exosomes are isolated and extracted from cell cultures, which is different from drugs or individual miRNA/lncRNA transcripts. This also provides a platform for engineering optimization. Now we know that the human CDC exosomes contain many miRNA/lncRNA transcripts that can improve fibrotic atrial remodeling, but they also harbor pro-arrhythmic transcripts (Vinciguerra et al., 2024; Zeng et al., 2023). Thus, straightforward engineering of producer cell lines to suppress or enhance pathological transcripts within EVs holds the potential to further augment the antiarrhythmic efficacy, necessitating further validation. Lastly, systemic therapy (e.g., intravenous or oral drug administration) may induce several avoidable adverse effects, including infection, gastritis, impaired wound healing, arrhythmia exacerbation, hyperglycemia, and bone marrow suppression. The identification of direct exosome injection into atrial myocardial tissue holds substantial significance and exerts a sustained effect, as this localized therapeutic approach is less prone to induce adverse effects in tissues distant from the target site, thus reducing the risk of off-target adverse effects and enhancing treatment safety.

We forecasted the interaction between XR_001793654.1 and miR-107-3p sequences utilizing miRanda software. miR-107 is part of the miR-15/107 cluster and exerts a substantial influence on human health and disease pathology (Turco et al., 2020). Members of the miR-15/107 cluster are among human expression miRNAs associated with cardiovascular disease. In a study involving patients with persistent AF on continuous vitamin K antagonist therapy, elevated miR-107 expression was found to correlate positively with other major cardiovascular adverse events in AF patients, except for stroke, as assessed by plasma miRNA levels (Rivera-Caravaca et al., 2020). However, the specific mechanism by which miR-107 impacts AF remains insufficiently explored.

Additionally, we determined that the interaction between XR_001793654.1 and miR-107-3p can ultimately exert biological effects by modulating the expression of the target gene KLF13. KLF13, a member of the KLF-like zinc finger protein family, is predominantly expressed in the heart. It has been indicated that KLF13 is a major mediator of glucocorticoid receptor signaling in cardiomyocytes and protects these cells from DNA damage and death (Meng et al., 2019). Furthermore, KLF13 influences the growth and differentiation of cardiomyocytes during cardiac development and morphogenesis (Cruz-Topete et al., 2016). Consequently, we hypothesized that KLF13 may be involved in atrial fibrosis during AF. Our findings demonstrated that the overexpression of miR-107-3p reduced KLF13 expression, whereas the silencing of miR-107-3p increased KLF13 expression. The dual luciferase assay confirmed that miR-107-3p could bind to the KLF13 3′UTR fragment. KLF13 exerts biological effects as a target gene of miR-107-3p. Moreover, we verified KLF13 expression in the left atrial appendage of patients with degenerative valvular disease and in a rabbit AF model. The results indicated that KLF13 expression was downregulated in the AF group. Masson staining further suggested that decreased KLF13 expression may contribute to AF atrial fibrosis.

Our findings confirmed that KLF13 was regulated through miR-107-3p, and the role of XR_001793654.1 in this regulatory process was also established. In the co-transfection experiment with AAV overexpressing XR_001793654.1 and AAV overexpressing miR-107-3p, overexpression of XR_001793654.1 could upregulate KLF13 expression, while the overexpression of miR-107-3p could partially counter this effect. Therefore, we deduced that the expression of XR_001793654.1 was declined in AF atrial myocytes, while miR-107-3p was elevated. Through the ceRNA mechanism, reduced XR_001793654.1 levels have diminished the capacity to competitively bind miR-107-3p, thereby potentiating the inhibitory effect of miR-107-3p on the target gene KLF13, down-regulating KLF13 expression, and promoting the occurrence and progression of AF atrial fibrosis.

This study has several notable limitations. First, preclinical animal models (e.g., rabbit models used in this study) are commonly employed to investigate the underlying mechanisms of AF. However, they are unable to completely replicate the complex pathophysiology AF and thus serve primarily as experimental models. Validation of EVs’ role in human AF is necessary to assess the clinical translational potential of these findings. Second, unlike real-world clinical scenarios, where patients frequently have pre-existing fibrosis and comorbidities (e.g., hypertension, diabetes), the rabbit AF models used in this study do not exhibit these pre-existing conditions. This limitation is especially important when evaluating the influence of EVs on pre-existing atrial remodeling in postoperative AF, which is influenced by factors such as advanced age, prior AF history, left atrial enlargement, hypertension and left ventricular dysfunction. As a result, the contribution of EVs to therapeutic outcomes may have been overestimated. Third, since the intramyocardial injection of EVs is performed during open heart surgery, the surgical intervention could be a confounder in the atrial fibrosis, inflammation and atrial function. This confounder was not assesed in the current study, which requires in-depth research in the future to elaborate. Last but not least, our criteria for diagnosing atrial hypertrophy mainly rely on the cross-sectional area of atrial muscle cells and the degree of fibrous tissue proliferation. Nevertheless, limitations in accurately quantifying this parameter were acknowledged due to the inherent challenges in controlling for the directionality and heterogeneity of fibrotic lesions. Future research should confirm whether the observed anti-fibrotic and anti-inflammatory impacts are applicable to models with pre-existing fibrotic remodeling—a key pathological feature of clinical AF.

## Conclusion

**FIGURE 7:**
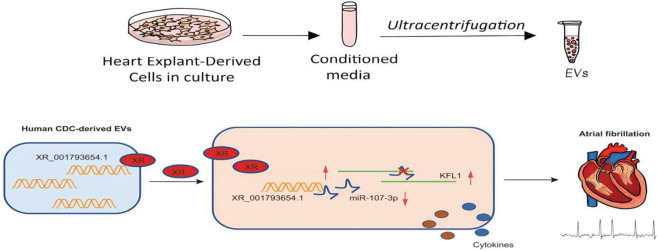
Schematic representation of the mechanism. EVs isolated from human CDCs carry XR_001793654.1, which binds to miR-107-3p and causes upregulation of the miR-107-3p target KLF13, thus alleviating AF.

To summarize, our research suggests that EVs derived from human cardiac explant cells (CDCs) are capable of transporting XR_001793654.1, a lncRNA that can bind to miR-107-3p and mitigate its inhibitory effect on the target gene KLF13, consequently reducing the impact of AF (Figure 7). As a result, the EV-mediated delivery of XR_001793654.1 could serve as a precise and responsive biomarker for the diagnosis and surveillance of AF progression.

## Data Availability

The raw data supporting the conclusions of this article will be made available by the authors, without undue reservation.

## Abbreviations

AF, atrial fibrillation; CDCs, cardiac explant-derived cells; EVs, extracellular vesicles; lncRNA, long non-coding RNA; miRNA, microRNA; RAP, rapid atrial pacing; LAD, left atrium diameter; LAVmax, left atrium maximal volume; LAVmin, left atrium minimal volume; LVEDV, left ventricular end diastolic volume; LVEF, left ventricular ejection fraction; LVESV, left ventricular end systolic volume.
