# Correction: Exosomal lncRNA XR_001793654.1 in human cardiac explant-derived alleviates atrial fibrillation via abolishing the miR-107-3p-mediated KLF13 inhibition

**DOI:** 10.3389/fcell.2026.1765022

**Published:** 2026-03-10

**Authors:** Yanxiao Liang, Dongyu Li, Huishan Wang, Yuan Tian

**Affiliations:** 1 Department of Cardiac Surgery, Shengjing Hospital of China Medical University, Shengyang, China; 2 Department of Cardiovascular Surgery, General Hospital of Northern Theater Command, Shengyang, China; 3 Department of Laboratory Medicine, Shengjing Hospital of China Medical University, Shengyang, China

**Keywords:** atrial fibrillation, extracellular vesicles, lncRNA XR_001793654.1, miR-107-3p, KLF13

There was a mistake in Figure 1D as published.

**FIGURE 1:**
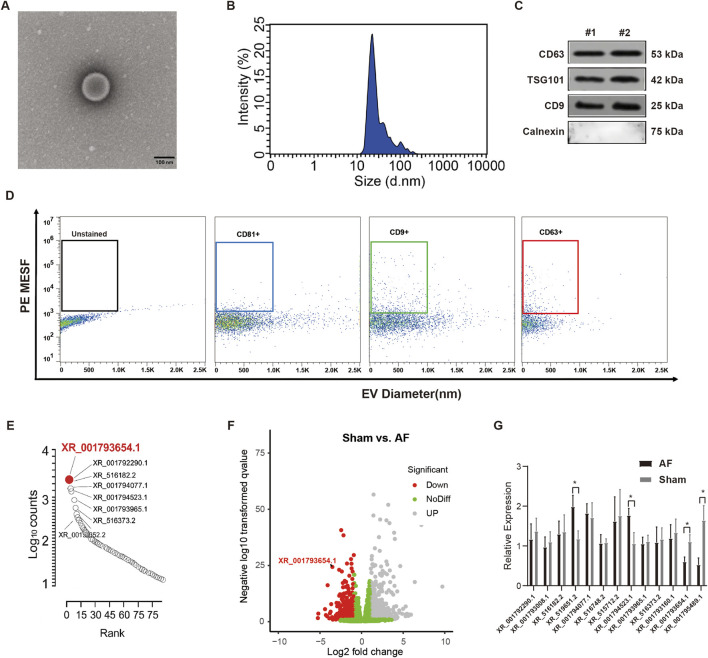
XR_001793654–1 exhibits high expression in the human CDC-derived EVs. **(A)** Transmission electron microscopy (TEM) for morphological analysis of EVs (scale bar = 100 nm). **(B)** Nanoparticle tracking analysis (NTA) for size distribution of EVs. **(C)** EV markers (CD63, CD9, TSG101) and the negative marker Calnexin were analyzed via Western blot. **(D)** Flow cytometric analysis of the size of vesicles and the expression of surface markers. **(E)** Quantification of lncRNA expression in human CDC-derived EVs. **(F)** Volcano plot of altered lncRNA expression in atrial fibrillation (AF) and sham rabbit models. **(G)** qRT-PCR analysis of atrial tissues from AF and sham rabbit models. *P < 0.05, #P < 0.01.

Due to the oversight in the file classification during the chart arrangement stage, presented in the original Figure 1D of the paper belong to the pre-experiment group’s experimental graph. The positive results presented are slightly worse than the results after optimizing the experimental operation. This error was a mistake in chart arrangement and organization.

This issue is merely a mistake in the presentation of the chart. It does not involve any changes to the experimental data collection, statistical analysis logic, or the core conclusions of the paper.

The corrected Figure 1 appears below.

The original article has been updated.

