# Supplementary material for: Exosomal lncRNA XR_001793654.1 in human cardiac explant-derived alleviates atrial fibrillation via abolishing the miR-107-3p-mediated KLF13 inhibition
Source: Front Cell Dev Biol. 2025 Nov 7;13:1694467. doi: 10.3389/fcell.2025.1694467 (PMC12634545; doi:10.3389/fcell.2025.1694467)
Supplement: Supplementary file 1 [file DataSheet1.docx]

**Supplementary Materials**

**Supplementary methods**

**Experimental model, skin preparation area, incision area**

In vivo study conduct and group sizes was shown in Figure S1. The scope of shaving and disinfection in the operation area was: left chest, upper abdomen and bilateral underarm. Remove residual hair with hair removal cream after shaving (Figure S2). Cardiac ultrasound was performed to measure left ventricular end-diastolic volume (LVEDV), left ventricular end-systolic volume (LVESV), left ventricular ejection fraction (LVEF), left atrial diameter (LAD), left atrial maximum volume (LAVmax), left atrial minimum volume (LAVmin), left atrial ejection fraction (LAEF) and other indexes.

**Position of stimulation electrode suture**

The auricle was carefully lifted with undamaged tweezers, and one stimulation electrode was sutured to the edge of the left auricle and the other to the top of the opposite left atrium, with the distance between the two electrodes ≥10mm (Figure S3).

**Stimulus pattern**

The stimulation system was turned on 3 days after the rabbit model resumed normal dietary activities, and the body surface electrocardiogram was continuously monitored. At the same time, two different stimulation modes were adopted to observe the tolerance of experimental rabbits, namely continuous stimulation and interval stimulation. The stimulation electrocardiogram was shown in Figure S4. The duration of routine stimulation is 2-4 weeks, and the stimulation is stopped if persistent spontaneous AF is present and sustained for more than 48 hours, and the stimulation is continued if no atrial fibrillation is present.

**Prestimulation and poststimulation electrocardiogram**

Normal electrocardiogram was observed on the operating system and the next surgical operation was performed after confirming that the electrocardiogram output was stable and the implanted pacemaker worked normally (Figure S5). During the operation of pacemaker implantation in model rabbits, the pre-stimulation signals, P wave, QRS wave group and T wave were clear and the baseline was stable. The program stimulation was started 72 hours after the postoperative recovery (the stimulation voltage of the continuous stimulation group was 2000 mV, the stimulation pulse width was 1 ms; In the interval stimulation group, the stimulation voltage was 2000 mV, the stimulation pulse width was 1 ms, the stimulation cycle was 50 ms, the stimulation was 2s, the pause was 2s). After stimulation, the heart rate of the rabbit model increased significantly, and slowly decreased after stopping the stimulation, indicating that the stimulation was effective.

**Structure and function of left atrium and left auricle**

The upper and lower diameters and left and right diameters of the left atrium were measured at the apical four-chamber section, and the anteroposternal anteroposternal diameters were measured at the level of the aortic valve in the parasternal long-axis section, and the volume of the left atrium was calculated (Figure S6). The maximum volume of the left atrium (LAVmax) and the maximum volume of the left atrial appendage (LAAVmax) were measured at the end of the T-wave, and the minimum volume of the left atrial appendage (LAVmin) and the minimum volume of the left atrial appendage (LAAVmin) were measured at the apex of the R-wave. Left atrial ejection fraction (LAEF) =(lavmax-lavmin)/LAVmax. Left atrial ejection fraction (LAAEF) =(LAAVmax-LAavmin)/LAAVmax*100%.

**Prestimulation and poststimulation cardiac uhrasonography**

According to the left atrial data measured by cardiac ultrasound, compared with the baseline data before stimulation, indicators such as left atrial internal diameter (LAD), left atrial maximum volume (LAVmax) and left atrial minimum volume (LAVmin) were significantly increased after stimulation, and left atrial ejection fraction (LAEF) was significantly decreased (P < 0.05) (Table S1 and Figure S7). Therefore, the rabbit model of rapid pacing induced atrial fibrillation can induce left atrial enlargement and dysfunction.

**Left Atrial and Left Auricular Structure and Function**

The upper and lower diameters, as well as the left and right diameters, of the left atrium were measured at the apical four-chamber view. The anteroposterior diameters were measured at the level of the aortic valve in the parasternal long-axis view, and the volume of the left atrium was calculated. The maximum volume of the left atrium (LAV_max) and the maximum volume of the left atrial appendage (LAAV_max) were measured at the end of the T wave, while the minimum volume of both the left atrium (LAV_min) and left atrial appendage (LAAV_min) was measured at the apex of the R wave. Left atrial ejection fraction (LAEF) was calculated as:


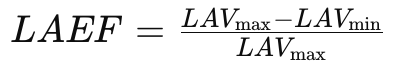


Left atrial appendage ejection fraction (LAAEF) was calculated as:


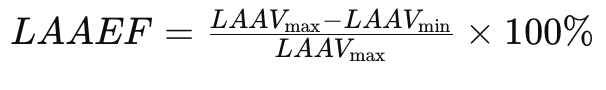


**Pre- and Post-stimulation Cardiac Ultrasound**
Based on cardiac ultrasound measurements of the left atrium, significant increases in the left atrial internal diameter (LAD), LAV_max, and LAV_min were observed post-stimulation compared to baseline measurements. Additionally, LAEF was significantly reduced (P < 0.05) (Table S2). These findings suggest that the rapid pacing-induced AF model led to left atrial enlargement and dysfunction.

**Masson’s Trichrome Staining**
Masson’s trichrome staining was performed on left atrial tissues from both stimulation groups to assess myocardial fibrosis. Collagen fibers stained blue, and cardiomyocytes appeared red. A comparison between normal atrial tissue and the stimulation groups revealed significant disorganization of atrial muscle tissue and fibrosis. No notable difference in fibrosis degree was observed between the interval and continuous stimulation groups.

**Histological Analysis and Fibrosis Quantification**
Atrial tissues were fixed and sectioned for histological examination of inflammatory infiltrates. Hematoxylin and eosin (H&E) staining, along with antibodies targeting activated T lymphocytes (CD3, ab16669, Abcam; CD4, ab237722, Abcam), macrophages (CD68, ab125212, Abcam; CD163, ab182422, Abcam), and neutrophils (CD11b, ab133357, Abcam), were used for immunohistochemical analysis. Inflammatory cell infiltration was quantified using the ImageJ color deconvolution plugin. Wheat germ agglutinin (WGA) was used to delineate cell boundaries. Atrial fibrosis was further verified by Masson’s trichrome and picrosirius red staining.

**Detection of Inflammatory Cytokines**
Atrial tissue was minced and homogenized using a tissue homogenizer. Inflammatory cytokines and chemokines were measured with a multiplex Luminex-based assay (LXSARM, R&D Systems), and each sample was analyzed in duplicate using a 96-well plate. IL-1β, IL-2, IL-18, and TNF-α were measured on a MAGPIX system (C4447b, Luminex). Data were processed using xPONENT software. Additionally, ELISA kits were used to quantify IL-6 (ERA32RB, Invitrogen), TGF-β1 (ab119558, Abcam), PDGF-AB (ab213906, Abcam), and MCP-1 (ab100778, Abcam) according to the manufacturer's instructions.

**Construction of lncRNA and miRNA Adeno-associated Virus (AAV)**The sequences for lncRNA XR_001793654.1 and ocu-miR-107-3p were synthesized by Hanheng Biotechnology Co., Ltd. The synthesized products were then inserted into the AAV expression vector for further use.

**AAV In Vivo Infection**
The experimental rabbits were anesthetized with a 3% sodium pentobarbital solution (30 mg/kg, administered via the ear vein). A multi-channel cardiac electrophysiology instrument and animal monitor were connected for real-time monitoring. Under aseptic conditions, the rabbit's neck skin was incised, and the subcutaneous tissue was bluntly dissected to expose the trachea, followed by endotracheal intubation. The respiratory settings were: frequency 30-40 breaths/min, tidal volume 25-30 mL, and a breath-to-exhale ratio of 2:10. The chest was opened along the left edge of the sternum to expose the left atrium. A multi-point oblique injection of 4 × 10^7^ transducing units (TU) of lentivirus was administered into the left atrial muscle to ensure even distribution. Following the procedure, the chest was closed, the skin sutured, and the surgical site disinfected and bandaged. Penicillin was intramuscularly injected for 3 days to prevent infection.

**Intramyocardial injection of EVs**

Intramyocardial injections were performed using a total volume of 100 μL injected using a Hamilton microsyringe (27-gauge needle) into the left atrial wall at 5 separate injection points. Injections into the atria were done as superficially as possible. During the injections, a bleb was visible under the epicardial surface, reminiscent of the small blister that forms under the skin when performing an intradermal injection. The needle was then retained at the site of injection for 5 seconds to prevent leakage from the site of injection. Prior to closing the chest, the edges of the pericardium were closely approximated but not sutured together. Additional doses of buprenorphine (0.03 mg/kg subcutaneous) were administered 6 and 12 hours postoperatively.

**Fluorescence Microscopy and qRT-PCR**
Four weeks after the procedure, the left atrial tissues were cryosectioned for fluorescence microscopy to evaluate AAV infection efficiency. Fluorescent signals confirmed successful virus transduction. qRT-PCR analysis of the left atrial tissue from the rabbit AF model injected with XR_001793654.1-overexpressing AAV (AAV-XR_001793654.1) revealed significantly higher lncRNA expression compared to the AAV-negative control (AAV-NC), confirming successful AAV infection.

**Immunohistochemistry (IHC) Staining**
Paraffin-embedded atrial tissues were sectioned at 5 μm. After antigen retrieval, the tissue sections were treated with 0.3% H2O2 for 10 minutes, blocked with 5% BSA, and incubated overnight at 4°C with the primary Mac-2 antibody (1:200). The sections were then re-immunostained with a biotinylated secondary goat anti-mouse IgG antibody (1:2500), followed by HRP-streptavidin incubation for 20 minutes. Development was performed with DAB, and counterstaining was done with hematoxylin. The images were captured using a Leica DM2500 microscope and analyzed with ImagePro Plus 7.1 software.

**RNA Sequencing (RNA-seq)**
Total mRNA was extracted from atrial tissues from each experimental group. The RNA was reverse-transcribed into cDNA using an oligo dT primer. The cDNA was then amplified and subjected to Tn5 transposase digestion to prepare the sequencing library. After passing quality control using an Agilent 2100 Bioanalyzer, the library was sequenced. Differential expression, GO, and KEGG pathway analyses were performed on the sequencing data.

Total RNA extraction was performed using TRIzol reagent. mRNA and lncRNA detection was carried out using reverse transcription kits, and miRNA was analyzed with a PolyA Tailing test kit. RT-qPCR was conducted using the SYBR Premix Ex Taq II kit on an ABI 7500 instrument, with U6 serving as a miRNA normalizer and GAPDH as the normalizer for other genes. Fold changes were calculated using the 2^-ΔΔCt^ method.

**Supplementary figures**


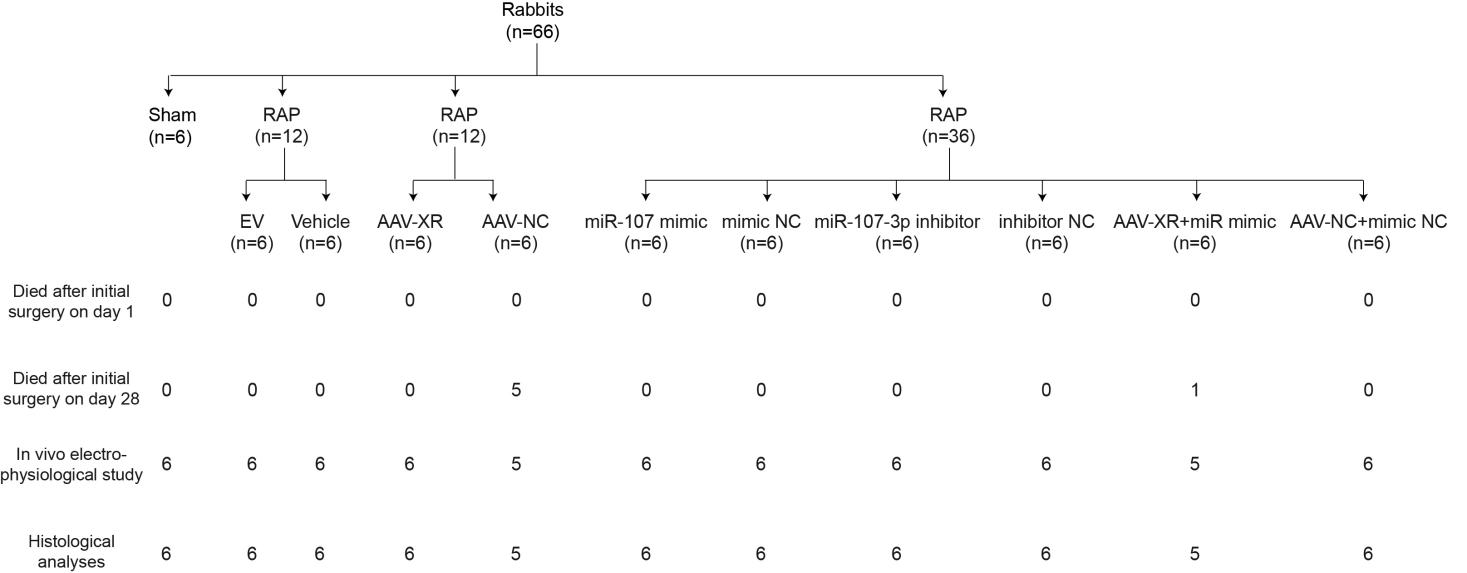


**Figure S1. In vivo study conduct and group sizes.**


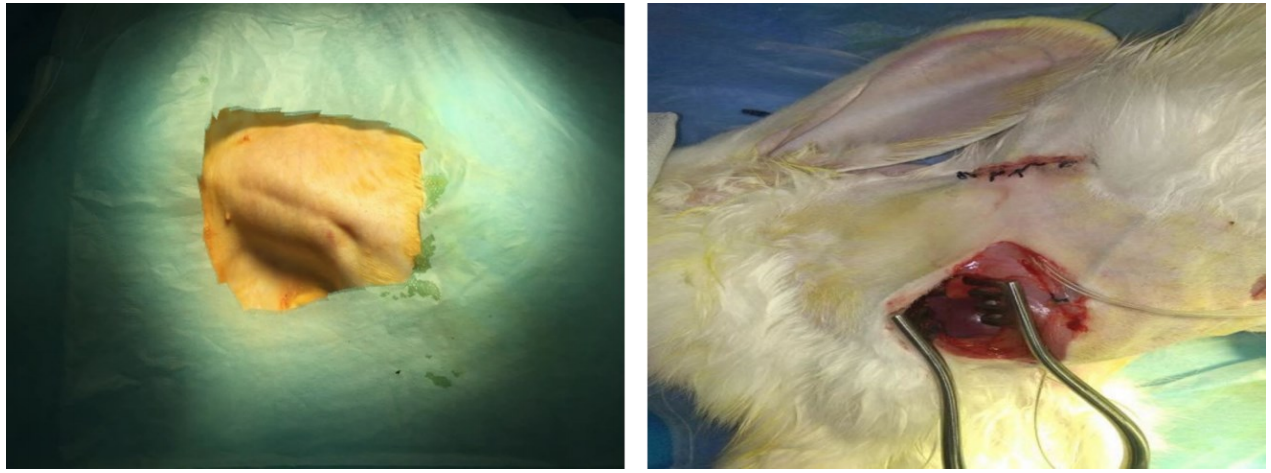


**Figure S2. Experimental model, skin preparation area, incision area**


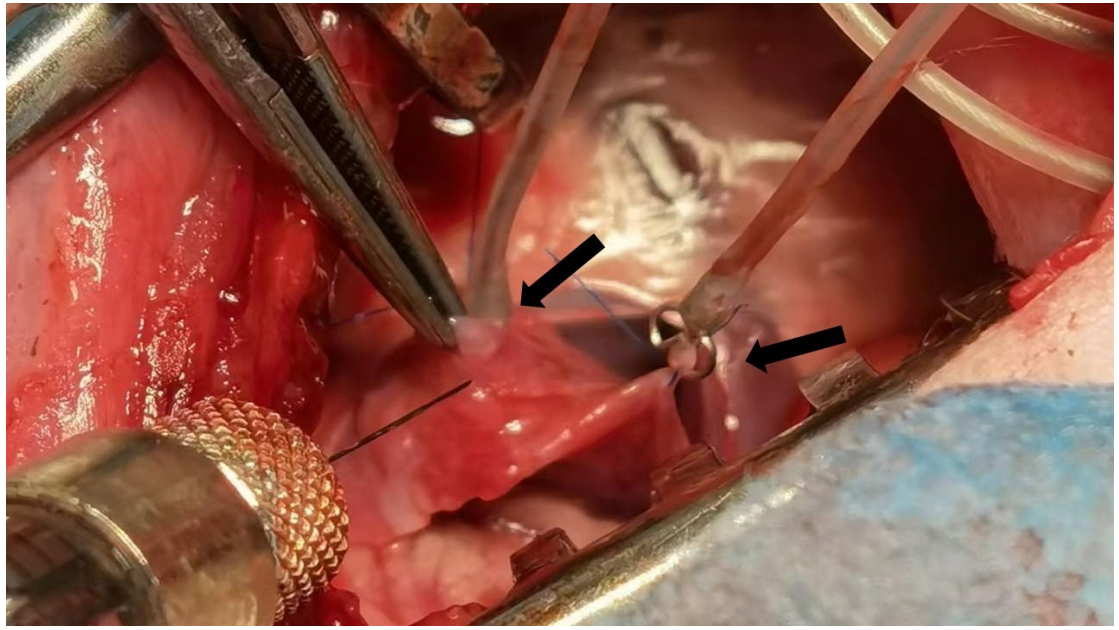


**Figure S3. Position of stimulation electrode suture**


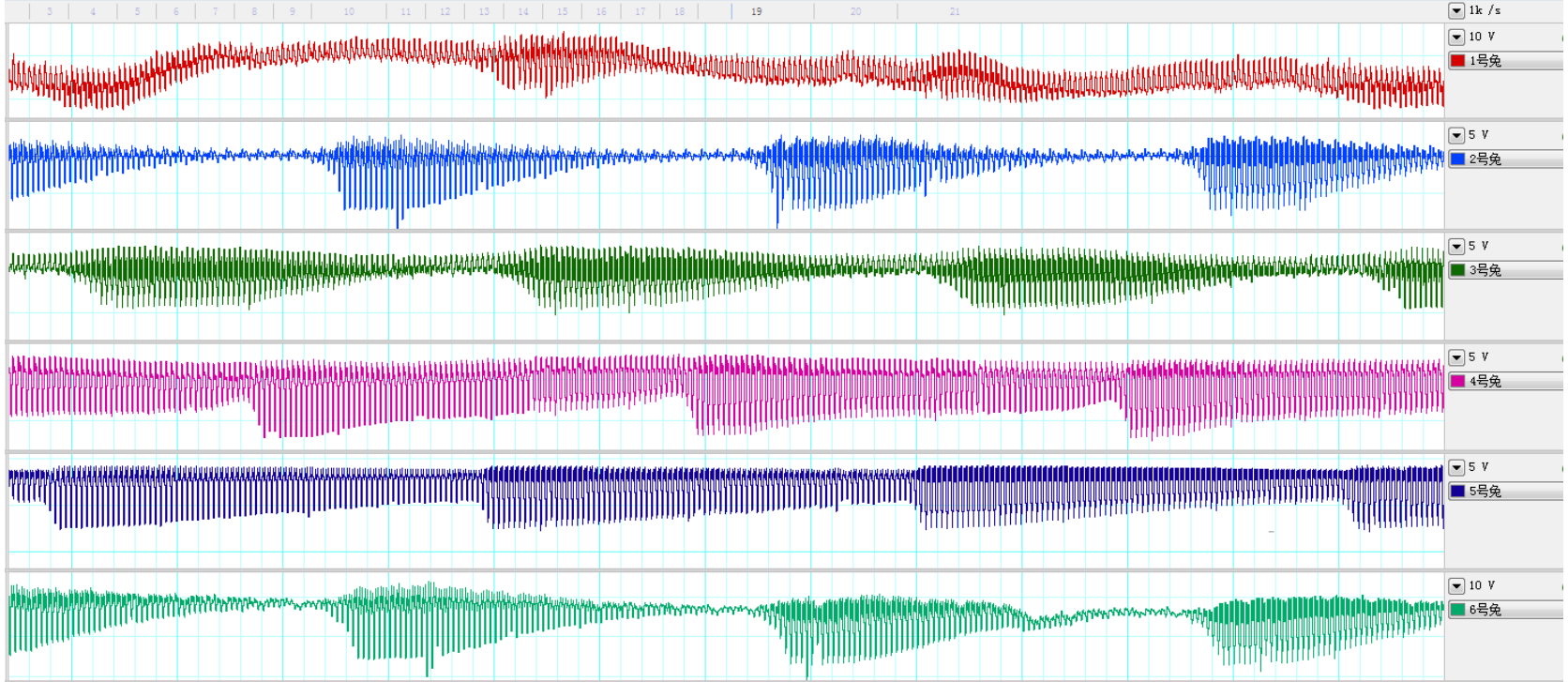


**Figure S4. Two different stimulation modes: channels 1, 4 and 5 were continuous stimuli; channels 2, 3 and 6 are interstitial stimuli**


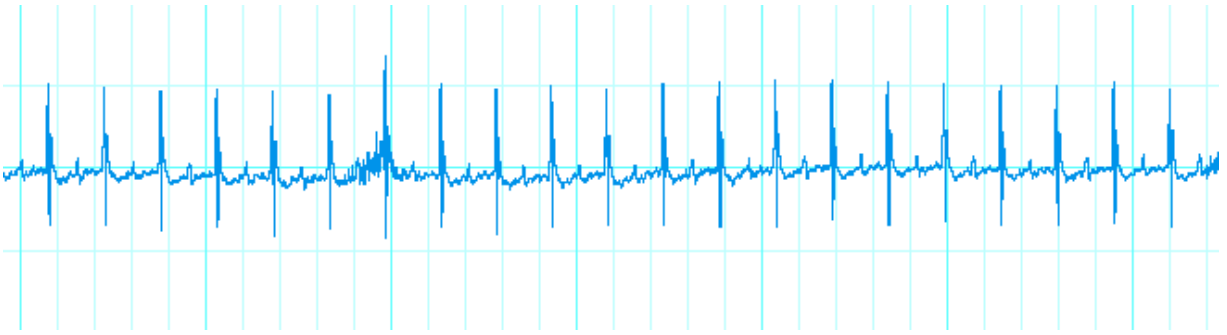


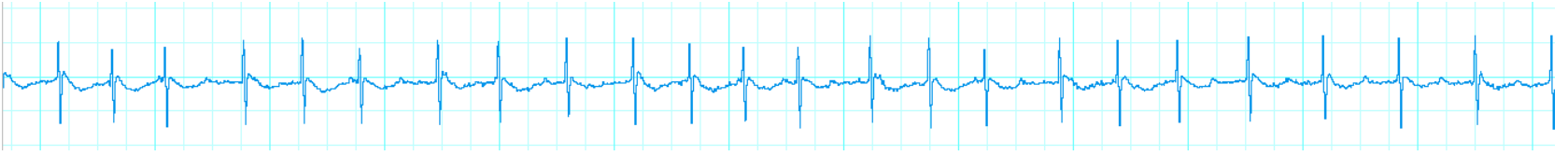


**Figure S5. Pre-stimulation and post-stimulation electrocardiogram**


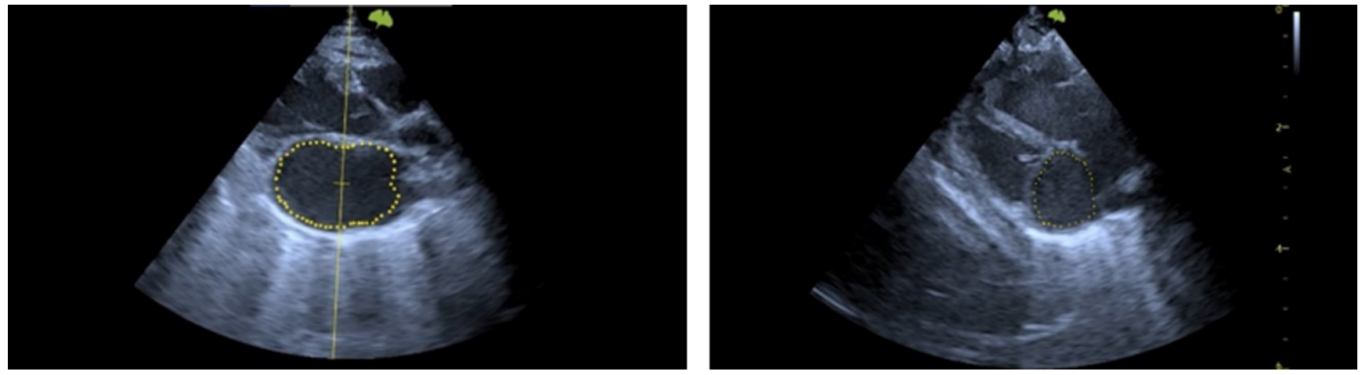


**Figure S6. Cardiac uhrasonography: parasternal long-axis section (left); apical four-chamber section (right)**


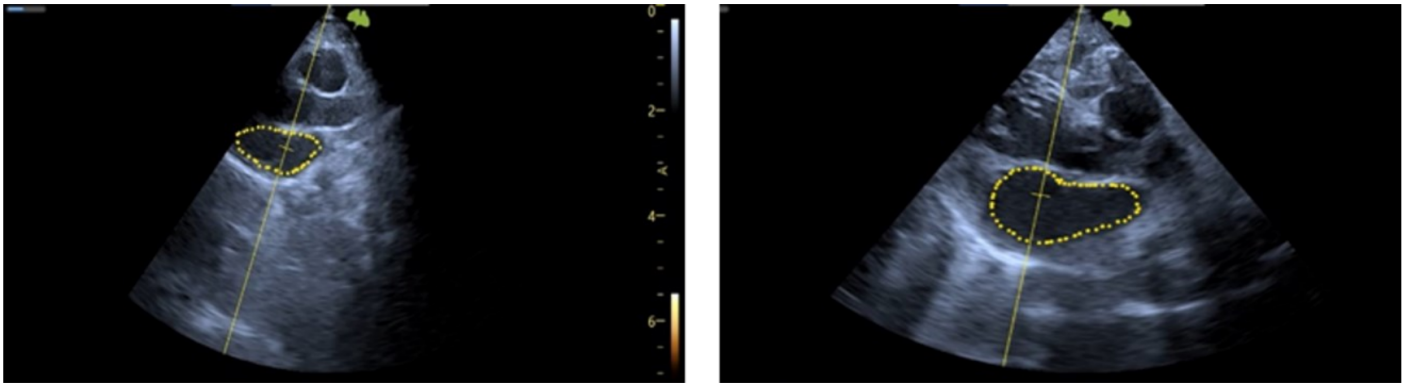


**Figure S7. Atrial fibrillation cardiac uhrasonography: pre-stimulus (left); post-stimulus (right)**


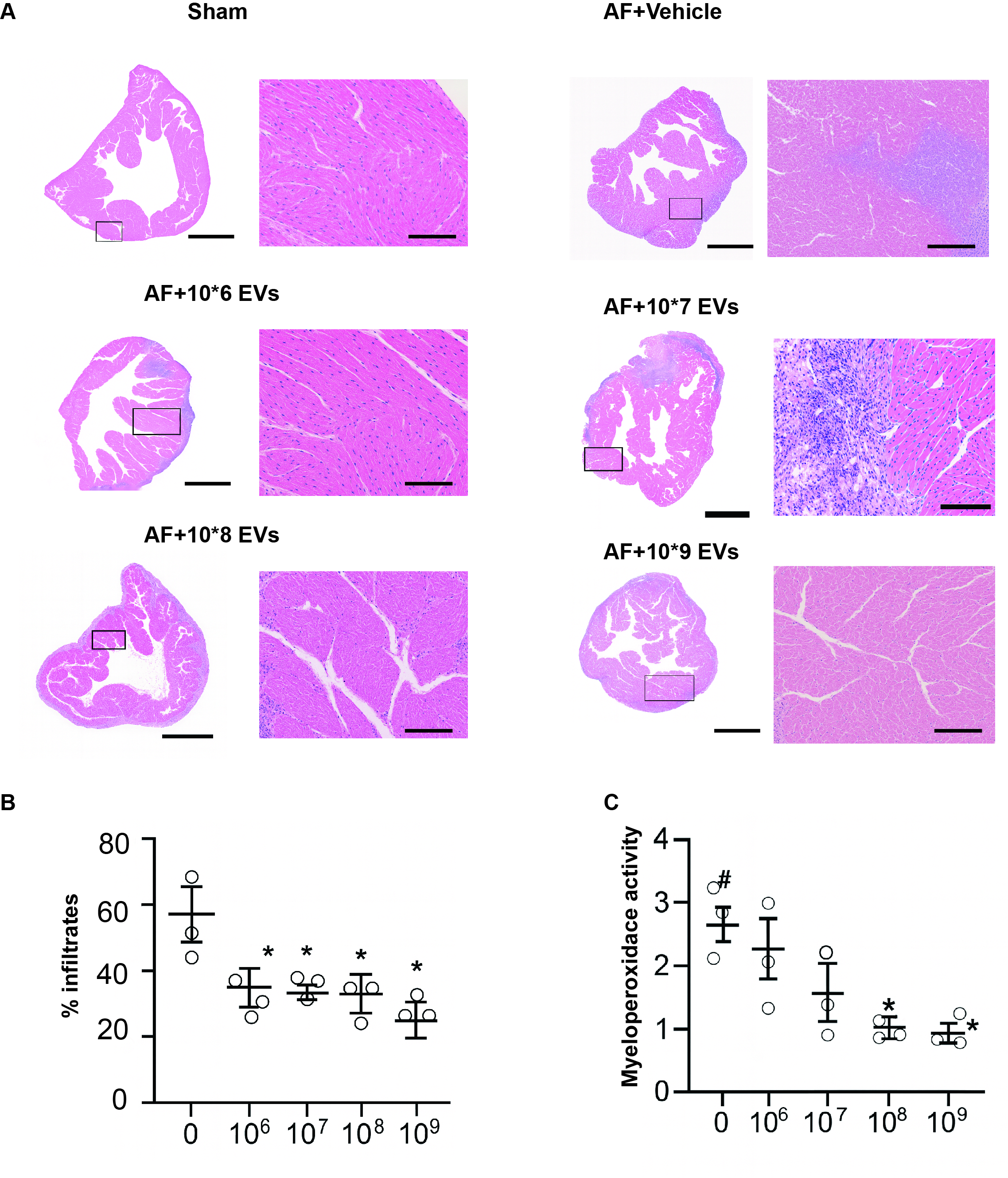


**Figure S8. Effect of increasing CDC EV doses on atrial fibrillation.**

1. Representative images from sham, AF+vehicle and AF+EV treated rabbits showing inflammatory infiltrates using H&E staining. Scale bar 2 or 0.2 mm, indicated. (B) Random field analysis showing that increasing EV dose had an immediate and progressive inhibitory effect on inflammatory infiltrates. Dotted line indicates baseline (sham). (C) Effect of EV treatment on myeloperoxidase activity. *P<0.05 vs. AF+vehicle treated animals. #P<0.05 vs. sham. EV, extracellular vesicles; AF, atrial fibrillation.


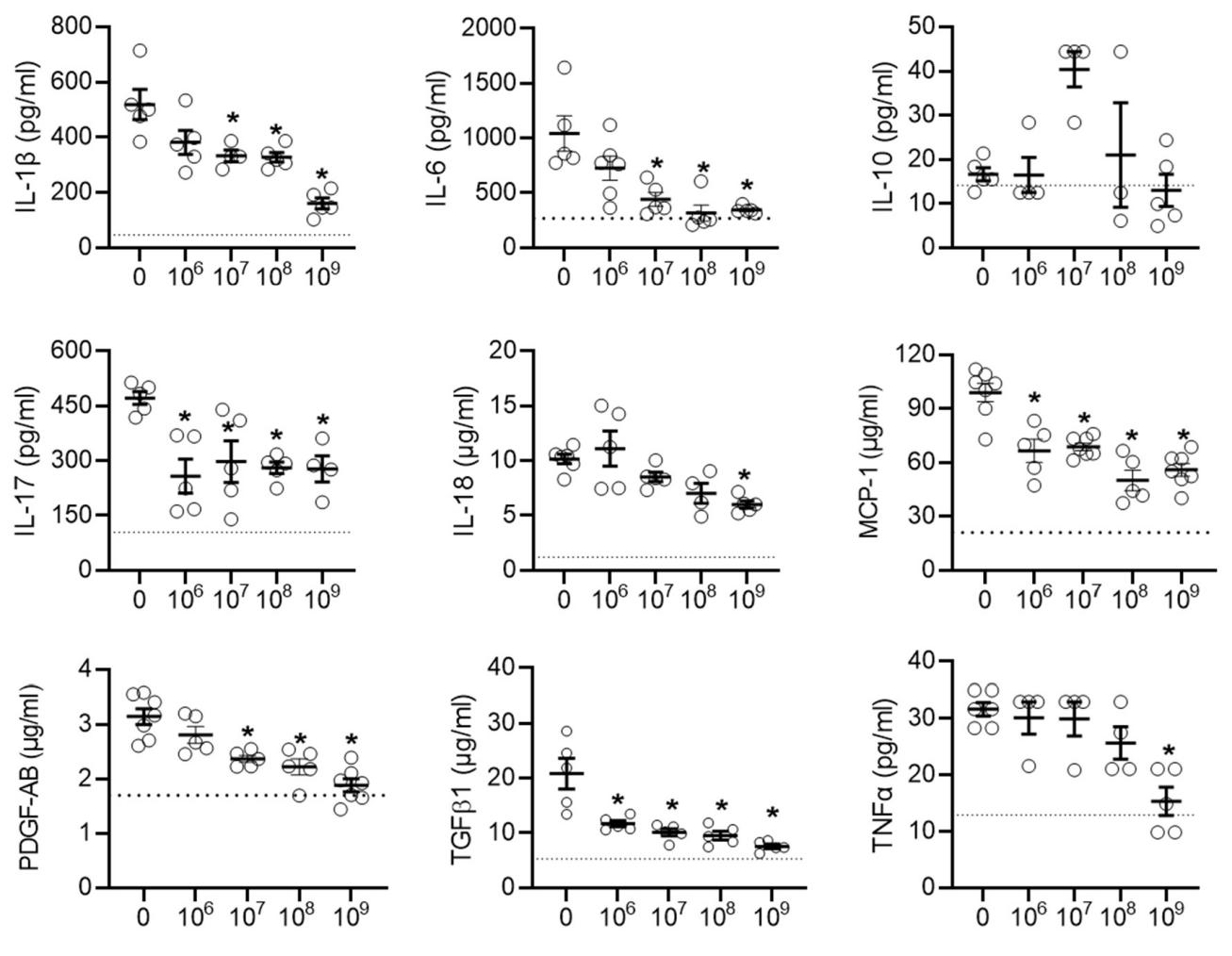


**Figure S9. Intramyocardial injection of CDC EVs reduced inflammatory cytokine content in a model of AF rabbit.**

Effect of increasing EV doses on cytokine levels within atrial tissue (n=5). *P<0.05 vs. AF+vehicle. IL, interleukin; monocyte chemoattractant protein 1, MCP-1; platelet-derived growth factor AB, PDGF-AB; transforming growth factor beta 1, TGFβ1; tumor necrosis factor alpha, TNFα.


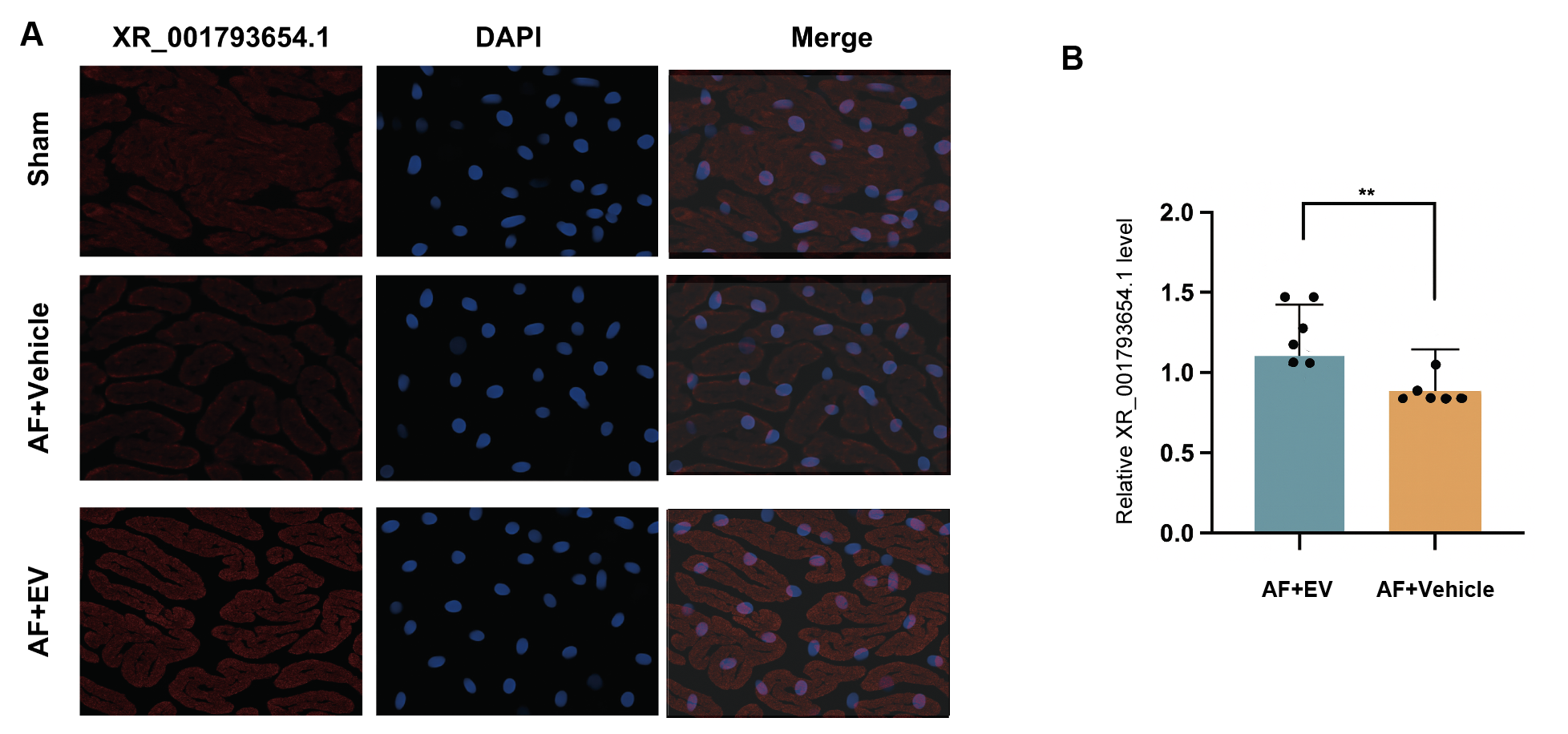


**Figure S10.** **EV-based lncRNA XR_001793654.1 transfer.**

(A) FISH shows increased XR_001793654.1 in cardiac slices of AF rabbits. (B) Relative expression levels of XR_001793654.1 in cardiac tisues of AF rabbits treated with or without EVs. **P < 0.01.


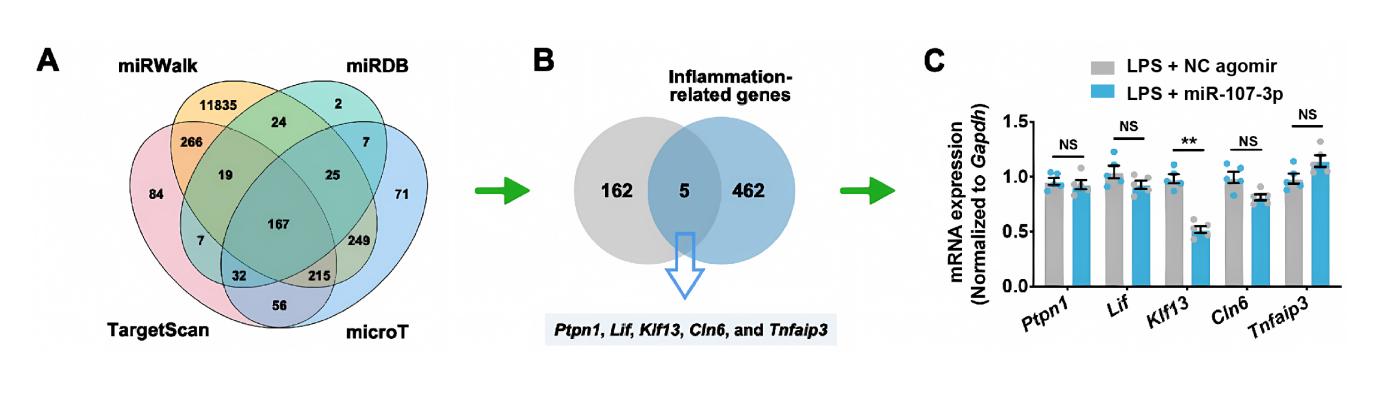


**Figure S11.** **Downstream mechanisms of miR-107-3p in regulating AF。**

(A) Four online databases (miRWalk, miRDB, TargetScan, and microT) were used to predict 167 candidate genes that may be targeted by miR-107-3p. (B) The 167 target genes predicted from online databases and 467 inflammation-related genes predicted from Disgenet were intersected to obtain five candidate genes. (C) The mRNA expression levels of Ptpn1, Lif, Klf13, Cln6, and Tnfaip3 were detected by RT-qPCR in in the left atrial appendage tissues of AF rabbits treated with miR-125a-5p agomir or NC agomir. **P < 0.01, NS: no significance.

**
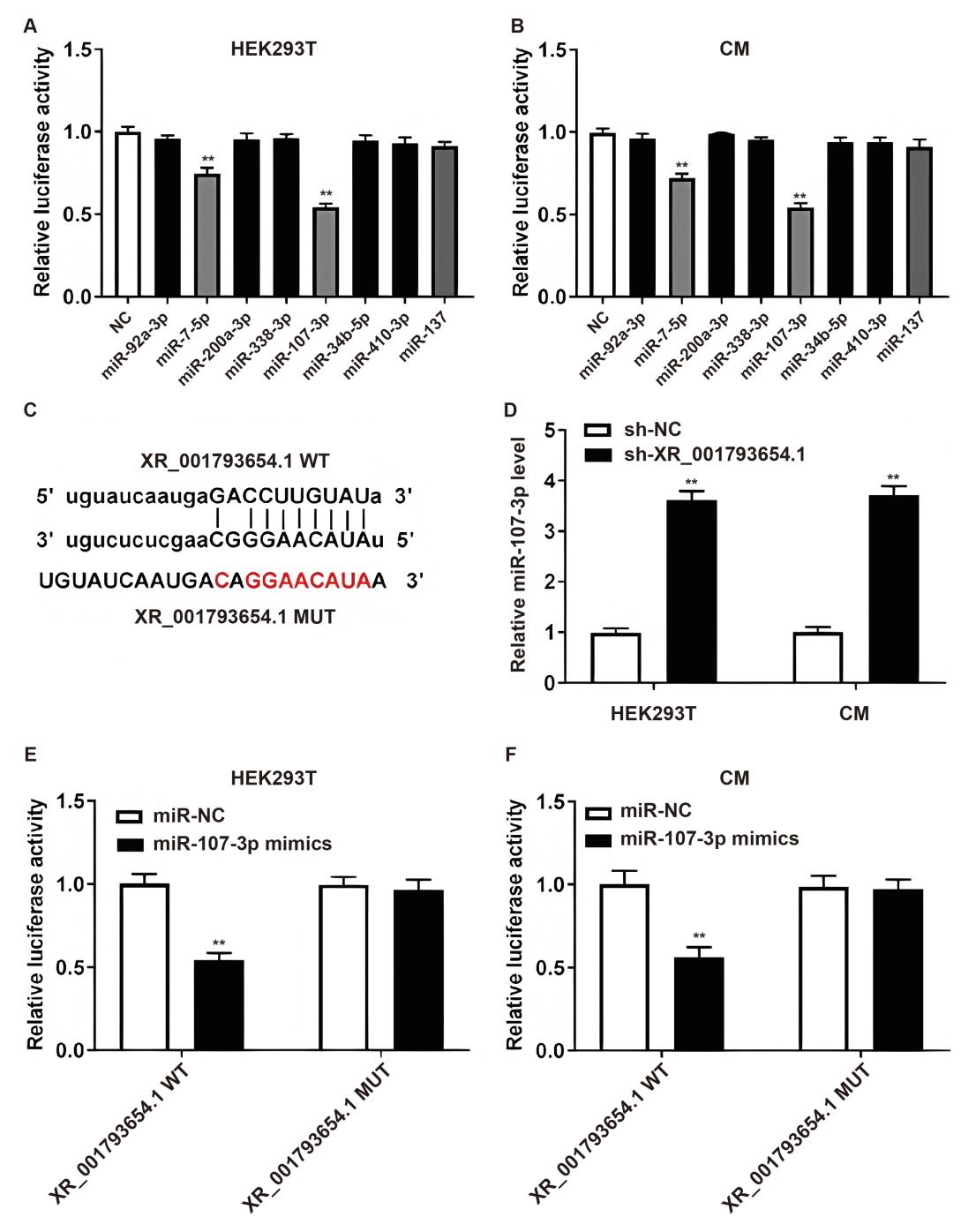
**

**Figure S12.** **LncRNA XR_001793654.1 targets miR-107-3p.**

(A, B) The luciferase activity of eight miRNAs selected from starbase database was detected by dual-luciferase reporter assay in both HEK293T and cardiomyocyte (CM) cell lines. **P < 0.01 vs the negative control (NC) group. (B) The predicted complementary binding site of XR_001793654.1 and miR-107-3p. (C, D) The expression of miR-107-3p after transfection of shRNA (sh)-XR_001793654.1/NC iwas detected by quantitative reverse transcription-PCR (qRT-PCR). **P < 0.01 vs the sh-NC group. (E, F) The luciferase activity in HEK293T and CM cell lines co-transfected with pGL3-XR_001793654.1 wild type (WT)/pGL3-XR_001793654.1 mutation type (MUT) and miR-107-3p mimics/NC was determined by dual-luciferase reporter assay. **P < 0.01 vs the miR-NC group.


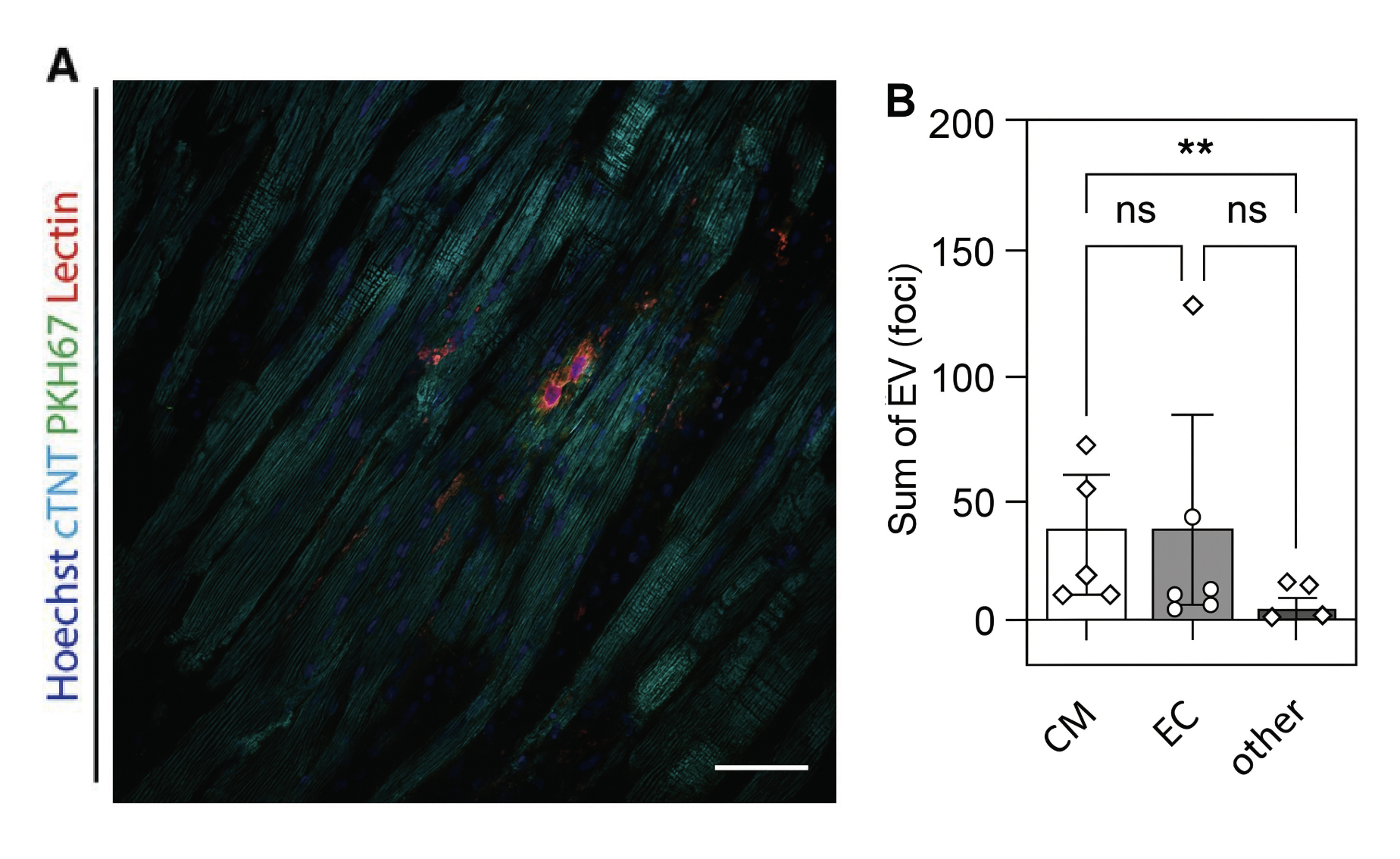


**Figure S13. EV uptake by the major cardiac cell types**

1. Representative confocal images of cardiac slices incubated for 24 h with PKH67-labeled EVs. Scale bar, 50 μm. White arrows indicate EVs taken up by a cardiomyocyte (CM), yellow arrows indicate EVs internalized by endothelial cells (ECs), and violet arrows indicate EVs internalized by other non-labeled cells (other). The imaged is magnified to allow the identification of EV foci in EC (top) and in CMs (bottom). (B) Sum of the EV foci that colocalize with a CM, with an EC, or with none of the prior cells (other) after 24-h incubation, demonstrating EV internalization by CMs, ECs, and other cells.


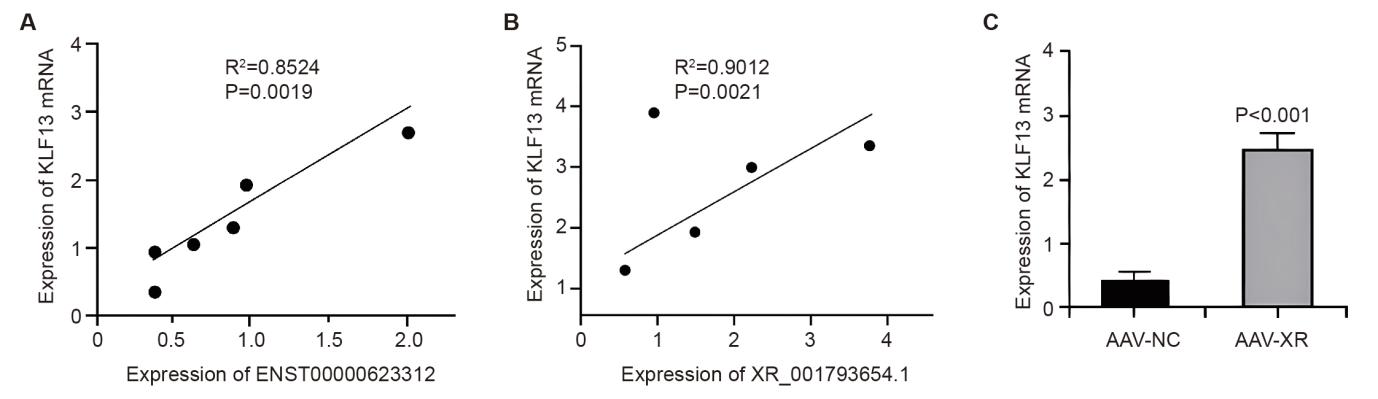


**Figure S14. Expression of XR_001793654.1 and KLF13 mRNA was positively correlated.**

(A) left atrial appendicular tissues of AF patients with degenerative valvular disease; (B) Left atrial appendicular tissues of rabbit AF model; (C) Expression of KLF3 mNRA after AAV-XR_001793654.1 or AAV-NC transfection.


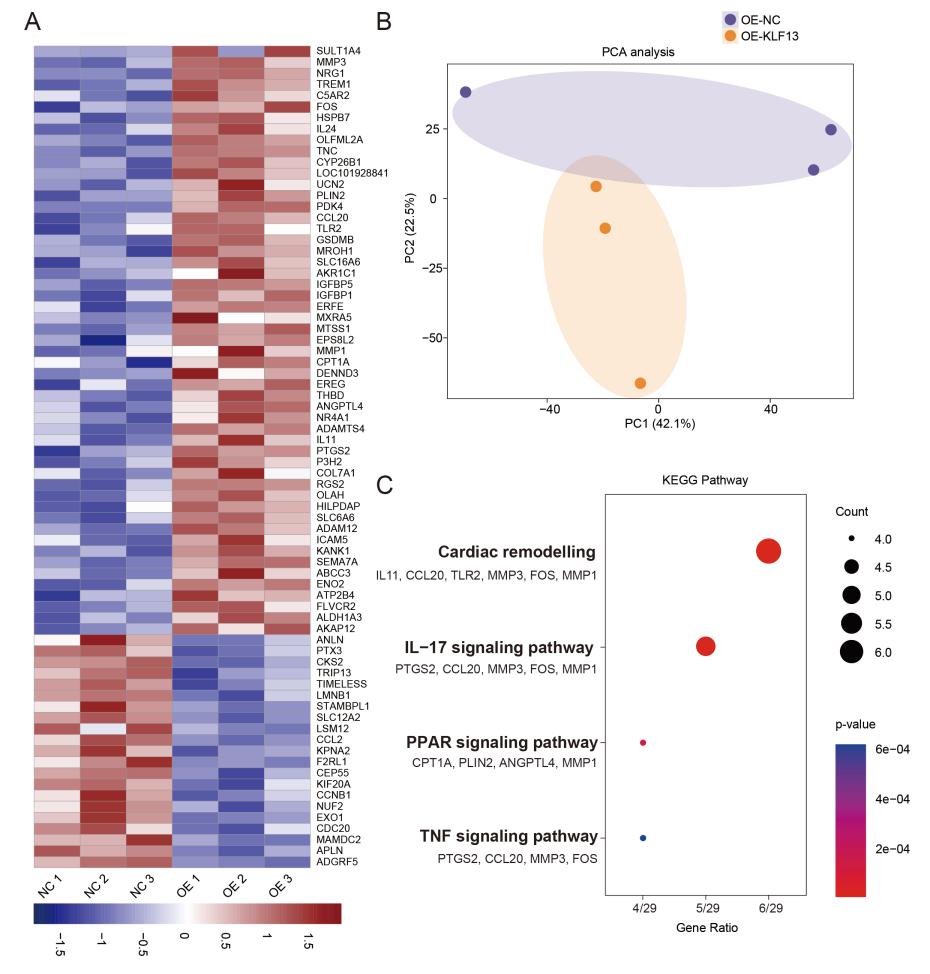


**Figure S15. KLF13 is involved in mediating anti-fibrotic and anti-inflammatory responses:** (A) Heatmap showing the dysregulated genes after KLF13 over-expression (OE) in human atrial fibroblast. (B) The Principal Component Analysis (PCA) showing the gene expression of KLF13. (C) The enriched KEGG pathways of dysregulated genes after over-expression of cKLF13.

**Table S1. Cardiac function measured by cardiac ultrasound.**

|  |  | LVEDV (ml) | LVESV (ml) | LVEF (%) | LAD (mm) | LAV_max (ml) | LAV_min (ml) | LAEF (%) |
| --- | --- | --- | --- | --- | --- | --- | --- | --- |
| continuous stimulation | baseline | 7.65±0.48 | 1.94±0.26 | 73.59±3.56 | 9.75±0.43 | 0.56±0.06 | 0.30±0.05 | 47.53±6.67 |
|  | after 4wk | 7.62±0.43 | 2.23±0.27 | 71.34±3.10 | 14.09±0.59* | 1.49±0.20* | 1.20±0.20* | 22.48±5.26* |
| interval stimulation | baseline | 7.54±0.45 | 2.16±0.25 | 70.28±4.48 | 9.59±0.38 | 0.51±0.05 | 0.34±0.01 | 45.08±8.30 |
|  | after 4wk | 7.42±0.34 | 1.90±0.30 | 73.14±2.76 | 13.89±0.60* | 1.35±0.12* | 1.14±0.09* | 22.90±3.84* |

**Table S2. Overexpression of XR_001793654.1 relieves the left atrial enlargement and left atrial dysfunction in rabbit AF model.**

|  |  | LAD (mm) | LAV_max (ml) | LAV_min (ml) | LAEF (%) |
| --- | --- | --- | --- | --- | --- |
| sham | baseline | 9.74±0.25 | 0.57±0.06 | 0.26±0.12 | 48.53±6.26 |
|  | after 4wk | 9.93±0.58 | 0.55±0.03 | 0.28±0.35 | 49.62±5.67 |
| AAV-NC | baseline | 9.83±0.37 | 0.54±0.06 | 0.27±0.04 | 47.93±7.84 |
|  | after 4wk | 14.89±0.61* | 1.48±0.21* | 1.20±0.19* | 22.45±3.31* |
| AAV-XR | baseline | 9.94±0.27 | 0.56±0.07 | 0.33±0.03 | 45.07±6.30 |
|  | after 4wk | 11.10±0.70* | 1.25±0.12* | 0.75±0.09* | 30.89±5.48* |

**Table S3. Clinical characteristics from AF patients undergoing cardiothoracic surgery.**

|  | Chronic AF | Paroxysmal AF |  |
| --- | --- | --- | --- |
|  | n = 15 | n = 12 | p value |
| Characteristics |  |  |  |
| Age, in years (mean ±SD) | 66 ± 12 | 71 ± 10 | 0.270 |
| Sex (male) (%) | 7 (47) | 8 (67) | 0.440 |
| Previous operation (%) | 4 (27) | 2 (17) | 0.660 |
| Hypertension (%) | 10 (67) | 9 (75) | 0.700 |
| BMI, in kg/m2 (mean ±SD) | 26.6 ± 4.2 | 26.1 ± 2.5 | 0.820 |
| Obesity (%) | 5 (33) | 1 (8) | 0.180 |
| Diabetes mellitus (%) | 4 (27) | 7 (58) | 0.130 |
| Insulin (%) | 1 (7) | 0 (0) | 1.000 |
| NIDDM (%) | 3 (20) | 7 (58) | 0.060 |
| Ischemic heart disease (%) | 4 (27) | 9 (75) | 0.020 |
| Renal impairment (%) | 3 (20) | 5 (42) | 0.400 |
| COPD (%) | 3 (20) | 0 (0) | 0.230 |
| Obstructive sleep apnea (%) | 1 (7) | 1 (8) | 1.000 |
| Dyslipidemia (%) | 7 (47) | 10 (83) | 0.110 |
| PVD (%) | 1 (7) | 3 (25) | 0.290 |
| Medication |  |  |  |
| Anticoagulation (%) | 15 (100) | 9 (75) | 0.070 |
| Statin therapy (%) | 9 (60) | 8 (67) | 1.000 |
| Echocardiographic |  |  |  |
| EF (%, mean ±SD) | 55.8 ± 6.9 | 48.7 ± 15.2 | 0.200 |
| LVEDD (mean ±SD) | 50 ± 8.9 | 50.2 ± 6 | 0.790 |
| LA diameter AP (mm, mean ±SD) | 52.7 ± 9.2 | 47.2 ± 7.3 | 0.070 |
| LA Systolic area (cm2, mean ±SD) | 36.1 ± 11.8 | 34.1 ± 12.2 | 0.740 |
| Moderate/severe MR (%) | 10 (67) | 4 (33) | 0.130 |
| Moderate/ severe MS (%) | 6 (40) | 1 (8) | 0.090 |
| Moderate/ severe AS (%) | 1 (7) | 4 (33) | 0.140 |
| Moderate/ severe TR (%) | 4 (27) | 3 (25) | 1.000 |
| SPAP (mean ±SD) | 47.1 ± 14.4 | 51.8 ± 25.3 | 1.000 |
| Surgery |  |  |  |
| Mitral valve repair/replacement (%) | 14 (93) | 6 (50) | 0.020 |
| Aortic valve replacement (%) | 2 (13) | 5 (42) | 0.180 |
| Tricuspid valve repair (%) | 7 (47) | 3 (25) | 0.420 |
| CABG (%) | 1 (7) | 8 (67) | 0.003 |
| Maze procedure (%) | 4 (27) | 3 (25) | 1.000 |

Abbreviations: AF = atrial fibrillation; AS = aortic stenosis; BMI = body mass index; CABG =

coronary artery bypass grafting; COPD = chronic obstructive pulmonary disease; LA= left

atrium; LVEDD = left ventricular end diastolic diameter; LVEF = left ventricular ejection

fraction; MR = mitral regurgitation; MS = mitral stenosis; NIDDM = non-insulin-dependent diabetes mellitus; PVD = peripheral vascular disease; SD = standard deviation; SPAP =

systolic pulmonary artery pressure; TR = tricuspid regurgitation.
